# ERDO - a framework to select an appropriate randomization procedure for clinical trials

**DOI:** 10.1186/s12874-017-0428-z

**Published:** 2017-12-04

**Authors:** Ralf-Dieter Hilgers, Diane Uschner, William F. Rosenberger, Nicole Heussen

**Affiliations:** 1 Department of Medical Statistics, RWTH Aachen University Aachen, Pauwelsstrasse 19, Aachen, Germany; 2 Department of Statistics, George Mason University, 4400 University Drive, Fairfax, 22030 VA USA; 3 Center of Biostatistics and Epidemiology, Sigmund Freud University, Freudplatz 1, Vienna, 1020 Austria

**Keywords:** Design, Restricted randomization, Selection bias, Chronological bias, Type I error probability

## Abstract

**Background:**

Randomization is considered to be a key feature to protect against bias in randomized clinical trials. Randomization induces comparability with respect to known and unknown covariates, mitigates selection bias, and provides a basis for inference. Although various randomization procedures have been proposed, no single procedure performs uniformly best. In the design phase of a clinical trial, the scientist has to decide which randomization procedure to use, taking into account the practical setting of the trial with respect to the potential of bias. Less emphasis has been placed on this important design decision than on analysis, and less support has been available to guide the scientist in making this decision.

**Methods:**

We propose a framework that weights the properties of the randomization procedure with respect to practical needs of the research question to be answered by the clinical trial. In particular, the framework assesses the impact of chronological and selection bias on the probability of a type I error. The framework is applied to a case study with a 2-arm parallel group, single center randomized clinical trial with continuous endpoint, with no-interim analysis, 1:1 allocation and no adaptation in the randomization process.

**Results:**

In so doing, we derive scientific arguments for the selection of an appropriate randomization procedure and develop a template which is illustrated in parallel by a case study. Possible extensions are discussed.

**Conclusion:**

The proposed ERDO framework guides the investigator through a template for the choice of a randomization procedure, and provides easy to use tools for the assessment. The barriers for the thorough reporting and assessment of randomization procedures could be further reduced in the future when regulators and pharmaceutical companies employ similar, standardized frameworks for the choice of a randomization procedure.

**Electronic supplementary material:**

The online version of this article (doi:10.1186/s12874-017-0428-z) contains supplementary material, which is available to authorized users.

## Background

Randomization is considered to be the most important tools to protect against bias and ensure the internal validity of a clinical trial, although this is intensively discussed, see [1]. The important objective of randomization is to have two groups that are as equal as possible, i.e., not favoring one side or the other. Many randomization procedures have been introduced to help mitigate bias, and their theoretical properties have been analyzed extensively [2]. Yet, most reports of clinical trials fail to indicate the randomization procedure used, and presumably have not considered its properties.

For example, the popular permuted block design is generally considered to reduce possible bias resulting from an unobserved time trend in the data. The toss of a fair coin on the other hand, also known as complete randomization, is assumed to mitigate bias resulting from conscious or unconscious selection of patients to the treatment groups. Clearly, both these randomization procedures have their advantages, but also their disadvantages.

During the planning stage of a clinical trial a scientist has to select one from a variety of possible randomization procedure as part of the design. Ideally, the choice is based on scientific arguments reflecting the special aspects of the trial setting. The ICH E9 guideline [3] recommends studying the potential contribution of bias to the *p*-value. In spite of this recommendation, neither the ICH guidelines nor the CONSORT statement [4] yield instructions to reach a scientifically guided decision on a particular randomization procedure.

It therefore appears that the selection of a randomization procedure is left to the scientist’s preference rather than on scientific arguments. This strengthens the misconception that any randomization, regardless of how it is conducted, is enough to reach validity. Consequently the reporting standard for randomization procedures in the literature is low [5]. Often one sees comments such as “randomization was done by Excel" or “randomization was done using a set of sealed envelopes", not recognizing the importance of the randomization procedure itself.

The deficit of a scientifically guided choice of a randomization procedure in the planning phase of a clinical trial may also be due to the fact that no standardly available software tools for a comparison study have been available–until now. Recent work has been published describing models [6–9] and a software tool [10] that facilitates comparisons of randomization procedures with respect to bias on the test decision.

The aim of this paper is to propose a structured template for the selection of a randomization procedure considering the impact of bias on the *p*-value. Focusing on two specific types of bias, we demonstrate that the influence of bias may be mitigated to a large extent by selecting an appropriate randomization procedure.

The first type of bias we consider arises from unobserved time trends due to heterogeneity across the chronology in patient responses [14]. Such time trends may bias the results of clinical trials; the resulting bias is referred to as *chronological bias* [15]. It can be shown that certain types of randomization procedures are less sensitive to chronological bias, and consequently hypothesis testing can be conducted with less concern about type I error rate inflation or deflation.

The second type is *selection bias*. Historically, selection bias has been interpreted as the intentional or unintentional selection of patients who may have a higher probability of responding to treatment. The first approach to quantify this bias goes back to Blackwell and Hodges [11]. Their model has been shown to be equivalent to a metric of the predictability of the randomization procedure [2], where predictability is defined as the difference between the conditional and unconditional allocation probabilities. As stated by Rosenberger and Lachin [2], all other considerations being equal a randomization procedure should be selected that is as unpredictable as possible to avoid selection bias. While some have argued that today’s multi-center clinical trials with centralized randomization are unlikely to suffer from selection bias, Berger [12] gives a number of examples where selection bias has had an important influence in a clinical trial. He further states that selection bias can lead to covariate imbalances and inflation of the type I error rate. This is particularly true in unmasked studies. Observing that one third of the orphan drug legislation in the European Union consist of open label studies [13], selection bias could likely to be an issue. Several authors specify a selection bias model that uses the size of the test as a measure of assessment of the impact of selection bias [6–9].

In this paper, a linked bias effect is introduced that incorporates both selection and chronological bias in the statistical model. The model is applicable to a continuous normal endpoint within a two arm parallel group design. The test distribution under model misspecification for no treatment effect is derived and two metrics are proposed to make decisions about the appropriateness of specific randomization procedures. Recently the *randomizeR* software [10] was released, facilitating these decisions by enabling the researcher to perform a scientific evaluation of randomization procedures. A classification of restricted randomization procedures is proposed resulting in a reasonably wide class of procedures to be considered.

For the **E**valuation of **R**andomization procedures for **D**esign **O**ptimization, the template “ERDO" is introduced. The template makes use of these new tools taking into account the specific aspects of the clinical trial under investigation, such as type of treatments connected to diseases, outcomes, designs, etc. The template follows Benda’s [16] more general template “clinical scenario evaluation". We illustrate the ERDO template using a case study that assesses the influence of potential selection bias and chronological bias on the type one error probability, enabling a scientifically informed choice of an appropriate randomization procedure.

The paper is organized as follows. First the theoretical background based on the study layout under consideration, i.e. a 2-arm parallel group, single center randomized clinical trial with continuous endpoint, with no-interim analysis, 1:1 allocation and no adaptation in the randomization process is introduced. This includes as main aspects the derivation of the distribution of the usual t-test statistic under misspecification as well as a new metric to reflect regulators “go-no-go” decision, i.e. the probability of sequences exhibiting a type I error probability less than or equal to 0.05. In the next section, the statistical model will be used to propose the ERDO, by introducing the corresponding template. We apply the ERDO template in parallel to a case study. In the last section we will draw some conclusions and discuss further aspects.

## Theoretical background

In this section the statistical model to plan and analyze a single center randomized clinical trial with a two arm parallel group design is introduced. The corresponding subsections refer to the statistical model, the model for selection bias as well as for chronological bias, the joint bias model and the metric.

### Statistical model

Let *y*_*i*_ be a continuous normal response of a patient *i*,1≤*i*≤*N*, within the two arm parallel group randomized clinical trial with total sample size *N*. Let the number of patients randomized to the experimental group (*E*) and to the control group (*C*), respectively, be denoted *N*_*E*_ and *N*_*C*_, so that *N*=*N*_*E*_+*N*_*C*_. The allocation is denoted by *T*_*i*_=1 if patient *i* is allocated to *E* and *T*_*i*_=0 if patient *i* is allocated to *C*. Further *τ*_*i*_,1≤*i*≤*N*, constitutes the fixed unobserved bias effect acting on the response of patient *i*. Several mechanisms leading to an unobserved bias effect are imaginable. The corresponding model can be written as

1 $$\begin{array}{@{}rcl@{}} y_{i} = \mu_{E} T_{i} + \mu_{C} \left(1-T_{i}\right) + \tau_{i} + \epsilon_{i}, \end{array} $$

where the errors are independent and identically distributed with common unknown variance *σ* ^2^ i.e. *ε*_*i*_∼*N*(0,*σ* ^2^),1≤*i*≤*N*.

### Model for selection bias

Selection bias can result from different mechanisms that depend on the practical circumstances of a clinical trial, see Berger [19]. We now specify the unobserved bias effect *τ* by a selection bias model relating the response to the allocation sequence. Randomization is supposed to mitigate the potential for selection bias in randomized clinical trials. Proschan [6] introduced a biasing policy based on the convergence strategy [11] and studied the impact of selection bias on the test decision based on the *z*-test; see also Kennes [7]. In a simulation study, Tamm [8] considered a more general biasing policy under the assumption of final balance, i.e. *N*_*E*_=*N*_*C*_=*N*/2, and used the *t*-test assuming homoscedasticity. To be more specific, let *N*_*E*_(*i*−1),*N*_*C*_(*i*−1) be the number of past treatment assignments to *E* or *C* after (*i*−1) assignments and *p*_*E*_(*i*−1)=(*N*_*E*_−*N*_*E*_(*i*−1))/(*N*−(*i*−1)) be the portion of remaining allocations to the experimental group *E* to the total number of remaining allocations to *E* or *C*. Then the biasing effect *τ*_*i*_ in model (1) takes the form 
2


where  denotes the indicator function taking values 1 if *x*∈*A* and 0 otherwise. The *selection bias effect* *η* is the amount of increase or decrease in the expected response. In other words, a patient with elevated expected response is allocated to the next treatment, which is supposed to be the preferred treatment 1 if the portion of remaining allocations to that treatment exceed a threshold *q*∈[1/2,1]. In the case *q*=0.5 the selection biasing policy can be rewritten as

3 $$\begin{array}{@{}rcl@{}} \tau_{i} = \eta\left(\text{sgn}(N_{E}(i-1) - N_{C}(i-1))\right), \end{array} $$

where the recruiting person introduces selection bias in the study results by selecting the allocation of the next patient based on knowledge or guessing of the previous assignments. The function sgn(*x*) takes the values 1,0,−1 depending on the sign of *x*. Tamm [8] showed by simulation, that *q*=0.5 in (2) is the worst case when permuted block randomization is used.

In practice, the specification of *η* might be a point of discussion. One technique is to define *η* as a of the treatment effect from the published data of a clinical trial. This technique can be used more frequently when clinical trials data become more readily available [20]. Another approach is to use an estimate of *η*, which can be derived from clinical trial data from a similar study. In the simple two arm parallel group design with continuous endpoint, where the main analysis can be conducted using a *t*-test, an additional linear regression variable can be included in the statistical model describing the selection bias policy. This results in a two way ANOVA model with main effects (treatment and selection bias) only. However, a similar approach can be used with more complicated models like ANCOVA by including an additional factor as main effect. Otherwise it may be sensible to choose *η* as a fraction of the effect size that was used in the sample size calculation.

### Model for chronological bias

Chronological bias may impact the results of clinical trials, in particular in rare diseases, where a long recruitment time may be related for example to changes in population characteristics, changes in diagnostic ability, or learning effects due to surgeon experience [18]. Tamm [15] proposed to use linear, stepwise or logarithmic shapes of the time trend. Using the notation above, the bias in model (1) can take one of the forms 
4


where the time trend effect *θ* is a positive number. It seems sensible to choose *θ* as a fraction of the variation in the data, i.e., the standard deviation or range.

### Model for joint additive bias

If both chronological and selection biases act together, a joint bias model is necessary. We propose an additive model to introduce both biases in (1) with

5 $$\begin{array}{@{}rcl@{}}  \tau_{i} = \underbrace{\theta \;\frac{i}{N_{E} + N_{C}} }_{linear\; time\; trend}+ \; \underbrace{\eta\; \text{sign}(N_{E}(i-1) - N_{C} (i-1))}_{selection\; bias \;with\; q=0.5} {\;.} \end{array} $$

Note that a multiplicative formulation can be used as well. Further, weighting of selection and chronological bias can be implemented via formulation of *θ* and *η*. As implied above, different formulations of time trend and selection bias can be incorporated, or a relaxed version of the selection biasing policy can be used.

### Metric

The ICH E9 guidance [3] states that “the interpretation of statistical measures of uncertainty of the treatment effect and treatment comparisons should involve consideration of the potential contribution of bias to the *p*-value, the confidence interval, or to inference”. It results the question how the “potential contribution of bias to the *p*-value” is measured for a randomization procedure. Usually, the type I error rate, i.e., the proportion of false positive test decisions, is considered, mostly by simulation. However, the contribution may vary from allocation sequence to allocation sequence, and the variability of the effect is worth consideration. We propose the new metric *the probability of sequences of a randomization procedure RP exhibiting a type I error probability ω less than or equal to 0.05* (*P*_*RP*_(*ω*≤0.05)). This is a quantity summarizing the impact over all randomization sequences and demonstrates the clinical consequences as well as the go-no-go decision of the regulator directly. We therefore propose to select the randomization procedure from a set of suitable randomization procedures showing the minimum value of the new metric *P*_*RP*_(*ω*≤0.05).

### t-Test under Misspecification

An analytical expression to calculate the type I error probability for each sequence under model (1) can be derived in the case of a two-arm parallel group design with continuous normal endpoint. Langer [31] derived a formula in the presence of selection bias only. This approach can be applied where only selection bias or only time trend bias or both are included in the evaluation study. Assume we are interested in testing the hypothesis of population means *H*_0_:*μ*_*E*_=*μ*_*C*_ using a *t*-test. With the notation above $\tilde {y}_{E} = \frac {1}{N_{E}}\sum _{i=1}^{N} y_{i} T_{i}$ and $\tilde {y}_{C} = \frac {1}{N_{C}}\sum _{i=1}^{N} y_{i} (1-T_{i}),$ the test statistic

6 $$ {\begin{aligned} S = \frac{\sqrt{\frac{N_{E} N_{C}}{N_{E} + N_{C}}} \left(\tilde{y}_{E} - \tilde{y}_{C} \right)} {\sqrt{\frac{1}{N-2}\left(\sum\limits_{i=1}^{N} T_{i} \left(y_{i} - \tilde{y}_{E}\right)^{2} + \sum\limits_{i=1}^{N} \left(1-T_{i}\right) \left(y_{i} - \tilde{y}_{C} \right)^{2} \right)}} \end{aligned}}  $$

follows a doubly non-central *t* distribution with non-centrality parameters

$$\begin{array}{*{20}l} \delta &= \frac{1}{\sigma} \sqrt{\frac{N_{E} N_{C}}{N_{E}+N_{C}}}\left(\mu_{E}-\mu_{C} + \tilde{\tau}_{E} - \tilde{\tau}_{C} \right)\\ \lambda &= \frac{1}{\sigma^{2}}\left(\sum_{i=1}^{N}\tau_{i}^{2} - N_{E} \tilde{\tau}_{E}^{2} - N_{C} \tilde{\tau}_{C}^{2} \right) \end{array} $$

under *H*_0_:*μ*_*E*_=*μ*_*C*_, where $\tilde {\tau }_{E} = \frac {1}{N_{E}}\sum _{i=1}^{N} \tau _{i} T_{i}$ and $\tilde {\tau }_{C} = \frac {1}{N_{C}}\sum _{i=1}^{N} \tau _{i} (1-T_{i})$. The unusual form of the model and test statistic is necessary to express the dependence on the allocation vector **T**=(*T*_1_,…,*T*_*N*_)^*t*^. The expectation of $\tilde {y}_{E} - \tilde {y}_{C}$ for a given allocation vector **T** is $ \mu _{E}-\mu _{C} + \tilde {\tau }_{E} - \tilde {\tau }_{C}$. Further, observing that the expectation of *y*_*i*_ is *μ*_*E*_ *T*_*i*_+*μ*_*C*_(1−*T*_*i*_)+*τ*_*i*_ and under the normal assumption of the error, the distribution $\sum _{i=1}^{N} T_{i}(y_{i} - \tilde {\tau }_{E})^{2} + \sum _{i=1}^{N} (1-T_{i}) (y_{i} - \tilde {\tau }_{C})^{2}$ is a non-central *χ* ^2^ with *N*_*E*_+*N*_*C*_−2 degrees of freedom and non-centrality parameter

$$\begin{array}{*{20}l} \lambda &= \frac{1}{\sigma}\left(\sum_{i=1}^{N} T_{i} \left(\tau_{i} - \tilde{\tau}_{E}\right)^{2} + \sum_{i=1}^{N} (1-T_{i})\left(\tau_{i} - \tilde{\tau}_{C} \right)^{2} \right)\\ &=\frac{1}{\sigma}\left(\sum_{i=1}^{N}\tau_{i}^{2} - N_{E} \tilde{\tau}_{E}^{2} - N_{C} \tilde{\tau}_{C}^{2} \right) \;. \end{array} $$

Thus, according to Johnson, Kotz, Balakrishnan (1995) [32], the statistic (6) follows a doubly non central *t* distribution with the above mentioned non centrality parameters. Using this distributional result, the two sided type I error probability under the null hypothesis *H*_0_:*μ*_*E*_=*μ*_*C*_ can be calculated from

7 $$ \begin{aligned} &P\left(|S|>t_{N_{E}+N_{C}-2}\left(1-{\alpha}/{2}\right) \big| \mathbf{T} \right)\\ &\quad= F \left(t_{N_{E}+N_{C}-2}\left(\frac{\alpha}{2}\right);N_{E}+N_{C}-2;\delta,\lambda\right)\\ &\qquad+ F\left(t_{N_{E}+N_{C}-2}\left(\frac{\alpha}{2}\right);N_{E}+N_{C}-2;-\delta,\lambda\right), \end{aligned}  $$

where *F*(*x*;*n*+*m*−2;*δ*,*λ*) denotes the distribution function of the doubly non-central *t*-distribution with *n*+*m*−2 degrees of freedom and non centrality parameters *δ* and *λ* and *t*_*n*+*m*−2_(*γ*) denotes the *γ*-quantile of the central *t*-distribution (*λ*=*δ*=0). In case of the biasing policy *τ*_*i*_=*η*(sign(*N*_*E*_(*i*−1)−*N*_*C*_(*i*−1))) the symmetry of the non-centrality parameters can be used to gain computational efficiency while evaluating the possible set of allocation vectors **T** of a particular randomization procedure. Formula (7) can be used to either calculate the two sided type I error probability for a specific allocation vector **T** or the expected two-sided type I error probability for a specific randomization procedure by summing over the whole range of allocation vectors **T**, weighted by the probabilities implied by the randomization procedure for the specific sequences. Obviously, giving the distribution of the type I error probabilities depending on the sequences induced by the randomization procedure provides more information than any summary measure. Consequently, this approach is recommended.

## The ERDO template

In this section, the concept of **E**valuation of **R**andomization procedures for **D**esign **O**ptimization (ERDO) is introduced. It is illustrated by a case study for the design of a particular randomized clinical trial. The steps of the evaluation process are given in the template (Table 1).

**Table 1:** ERDO template

1	Introduction and objective
2	Framework
	(a)Assumptions
	(b)Options
	(c)Metrics
3	Evaluation methods
4	Software
5	Results
6	Discussion and conclusion

We now describe the six steps of the template in detail illustrated by the *EnBand* case study. In any clinical trial for which this template is implemented, an appropriate section in the statistical analysis plan of the study protocol should be added.

### Step 1. Introduction and Objective

For the first step, the problem of selecting the appropriate randomization procedure should be described, taking into account the particular situations unique to the clinical trial. At the end of the step, a clearly defined objective should be stated. Of course, the objective in our particular setting is the selection of a randomization procedure for a particular clinical trial. Reference to the specifications of the study and to the objective of the evaluation of the randomization procedure should be given accurately.

#### **Case Study (EnBand-Study)**

An international multicentre clinical trial (SPR-Study) [17] compared scleral buckling (SB) with primary pars plana vitrectomy (PPV) in rhegmatogenous retinal detachment. The study could not answer whether an encircling band improves one year best corrected visual acuity in pseudophakic eyes of the scleral buckling group. Thus we would like to design a new clinical trial to investigate the effect of an additional use of an encircling band on one year best corrected visual acuity, called the “EnBand-Study”. We recognize that in almost all surgical trials learning effects can be assumed, which may induce chronological bias [18]. Of course, open label treatment allocation is related to the comparison of two surgical procedures, which may introduce selection bias. Thus both biases should be considered in the selection of a randomization procedure.

The evaluation of randomization procedures for clinical trial design “optimization” has as its objective determining an appropriate randomization procedure for the EnBand-Study with respect to selection and chronological bias. To this end, we will use the supporting information provided by the SPR-Study.

### Step 2: Framework

*2(a) Assumptions* In this subsection, we recommend describing the study layout, the statistical model, the types of bias and the metric to measure these biases in detail. Usually, there are many design aspects which have to be taken into account, such as the result of the sample size calculation including the defined effect size, the desired allocation ratio, the type of endpoint, the layout of the study, the number of treatment arms, stratification factors, and the number and the timing of interim inspections.

#### **Case Study (EnBand-Study)**

(continued). The EnBand-Study will be designed as a two arm parallel group single centre randomized clinical trial with continuous endpoint, no interim analysis and a 1:1 allocation ratio as well as no adaptation in the randomization process. The study endpoint best corrected visual acuity is measured as the log of the minimal angle of resolution (MAR), which may be considered continuous. The treatments cannot be masked since it is a surgical procedure. Based on the results of the SPR-Study in the SB group, a mean change in visual acuity of 0.52 (SD 0.77) was observed in the encircling band and 0.90 (SD 0.73) was observed in the group without encircling band. The resulting effect size is 0.497. To show this difference using a two-sided *t*-test at a significance level of 0.05 and a power of 80% with the pooled standard deviation 0.765, at least 65 patients per group are necessary.

It is decided to use an 1:1 allocation ratio, with a fixed sample design. Further, although the study will be conducted as a multicentre clinical trial, the treatment difference and the effect size are assumed to be homogeneous in all centres. The null hypothesis of no difference in the best corrected visual acuity after one year between the groups PPV with encircling band and PPV alone, will be tested with a *t*-test assuming equal variances.

We used the data of the pseudophakic sub-trial of the SPR-study to estimate the potential magnitude of selection bias. Based on the experience of the SPR-study selection bias may occur for various reasons in the EnBand-study. Although during the setup of the SPR-study a lot of emphasis was given to a unique interpretation of the inclusion criteria it shows up that this was in practice difficult to achieve. This weakness in the formulation of the inclusion criteria, e.g. “medium severity” may cause the potential for selection bias to a certain amount. Further, this is supported by the fact, that the surgeon decide about enrollment of the patient intra operatively, when some of the inclusion criteria are determined. Randomization was implemented by sealed opaque envelops. As a consequence, in 10 cases intra operative treatment crossovers were observed. We estimated an selection effect *η*=0.09 as described above. Further, from the residual sum of squares we derived the corresponding standard deviation *σ*=0.73 using the 243 uncorrected total degrees of freedom. It is convenient to standardize the selection bias effect, which yields *γ*=*η*/*σ*=0.12. Another approach for calculation of *η*,*σ* and *γ* is presented by Kennes et al. [21] using a bias-corrected test based on the maximum likelihood estimates. This leads to the same results.

**Fig. 1:**
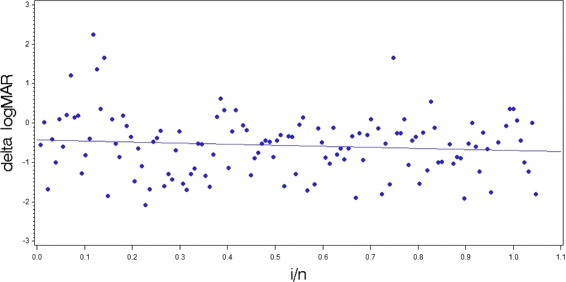
Scatterplot of change in best corrected visual acuity of the pseudophakic patients treated with scleral buckling of the SPR-study

We used the data of of the pseudophakic patients treated with scleral buckling of the SPR-study to predict the time trend of the EnBand-study and detected to a linear time trend of the magnitude 0.26 *i*/*n*, see Fig. 1.

In summary, to determine the best practice randomization procedure for the design of the EnBand-Study, the joint additive bias model (5)

$$\begin{array}{@{}rcl@{}} \tau_{i} = 0.26 \; \frac{i}{65 + 65} + \; 0.09 \; \text{sign}(N_{E}(i-1) - N_{C} (i-1)) \end{array} $$

is used, assuming the estimated standard deviation *σ*=0.73.

*2(b) Options* In this section, we propose to specify the randomization procedures under evaluation while taking into account their parameterization and specific properties. A comprehensive review of the randomization procedures is given in Rosenberger and Lachin (2016) [2], so we do not repeat the details of their computation here.

In order to choose the randomization procedure which best mitigates bias, we recommend including a variety of procedures in the evaluation, covering the whole spectrum of available procedures. We identify three partly overlapping classes of randomization procedures that arise from different types of restrictions imposed on the randomization process. We now introduce the classes proceeding from the weakest to the strongest restrictions.

We start with the class of randomization procedures where weakest restrictions are imposed. *Complete randomization* is within this class and is characterized by unrestricted treatment assignments without any control of the imbalance neither during nor at the end of the trial. The procedure is accomplished by tossing a fair coin, so the probability that patient *i* will receive treatment *E* is always 1/2, and may be considered as the “gold standard" with respect to unpredictability. Most clinical researchers avoid complete randomization because it can lead to large imbalances on the number of patients on each treatment either at the end or during the course of the trial, especially in small samples. Another candidate is *Efron’s biased coin design* [22] (EBC(*p*)) which consists of flipping a biased coin with probability *p*≥0.5 in favor of the treatment which has been allocated less frequently, and a fair coin in case of equality. Note that this class includes complete randomization (CR) when *p*=0.5. With Efron’s biased coin more unbalanced allocation sequences become less probable. The third candidate in this group is *Wei’s urn design* [24] (UD(*α*,*β*), where *α* and *β* are user specified nonnegative integer parameters. The procedure tends to balance treatment assignments by adaptively modifying the next allocation probabilities based on the current degree of imbalance. It can be regarded as an adaptively biased coin design.

One restriction implemented in randomization procedures is to control the imbalance during the treatment assignment process. Randomization procedures which ensure that the difference in the number of treatment assignments does not exceed a certain value either exact or by probability during the allocation are designed to control a given *maximum tolerated imbalance* [23]. A procedure which controls the imbalance strictly is the *big stick design* [25] (BSD(*a*)), which can be implemented via complete randomization with a forced deterministic assignment when a maximal imbalance *a* is reached during the enrollment. Another candidate related to Efron’s biased coin is *Chen’s design* (Chen(*a*),*p*) [26], where a maximum tolerated imbalance is applied to Efron’ biased coin. A broader class of designs results from the *accelerated biased coin design* [27]. The *maximal procedure* of Berger (MP(*a*)) is another candidate, which, in the most recent version, controls the maximal tolerated imbalance, but does not force balance at the end of the allocation process [28].

The next type of restriction is characterized by controlling the total imbalance after completion of the assignment process. Randomization procedures which ensure that the difference in the number of treatment assignments does not exceed a certain value at the end of the allocation process control the *final imbalance*. One candidate is *Random allocation rule* (RAR), which assigns half the patients to *E* and *C* randomly. *Permuted block randomization* (PBR(b)) with block size *b* uses RAR within blocks of *b* patients, and therefore controls the maximum tolerated imbalance as well as terminal balance.

For the evaluation and comparison of randomization procedures, some candidates are natural choices, such as CR which is considered gold standard for unpredictability, and RAR and PBR for a strict control of the imbalance during and at the end of a trial. Note that the permuted block design is the most frequently used procedure [28]. It is the investigator’s decision which procedures to include in the comparison study. However, due to the different properties we strongly recommend including at least one representative from each class in the evaluation study. For small trials, the use of complete randomization is not suitable as it does not control any imbalances, and can therefore lead to a loss in power. For example, for total sample size *N*=50, the probability for an imbalance of 25% that leads to a loss in power of 5% (reduction from 80 to 75%) is larger than 3%.

#### **Case Study (EnBand-Study)**

(continued). In the case study we include Efron’s biased coin with Efron’s suggested probability of *p*=2/3=0.67, Berger’s maximal procedure, the Big-Stick Design and Chen’s design which controls the maximum tolerated imbalance strictly and Wei’s urn design which controls the maximum tolerated imbalance adaptively are considered. As representatives of the class to control the final imbalance, the random allocation rule is included in the case study, as well as the permuted block design, which controls both maximum tolerated and final imbalance.

*2(c) Metric* The application of the ERDO requires a suitable metric for the target criterion that reflects the objective of the evaluation. A large number of different metrics have been defined in the literature, such as the expected number of correct guesses [11], the loss in power [2], or the balancing behavior [29]. Less work has been done to combine the different metrics. For instance, Atkinson [29] investigated the loss in power by imbalance and the impact of bias by the the average number of correct guesses. Schindler [30] proposes a unified linked assessment criterion to combine various standardized metrics. In the case study, we considered the value of the new metric *P*_*RP*_(*ω*≤0.05) as well as for comparative reasons the mean type I error probability.

#### **Case Study (EnBand-Study)**

(continued). For the EnBand-Study, the derivation above can be used to evaluate the randomization procedures with respect to the probability of sequences exhibiting a type I error probability less than or equal to 0.05. This metric fulfills the ICH E9 [3] recommendation to study the potential contribution of bias with respect to the *p*-value acceptably.

### Step 3. Evaluation Method

For this step, we recommend a concise description of the method used for the evaluation of the randomization procedures. Usually it will comprise a comprehensive simulation study rather than analytical results. Different randomization procedures should be considered with varying parameter settings (e.g., different block sizes, in case of the permuted block design or different values of *p* in Efron’s biased coin design). As mentioned in step 2(b), the set of randomization procedures under evaluation should be large and diverse with respect their properties.

The estimates of the selection bias effect *η* and the time trend effect *θ* should be derived from the literature or preceding clinical trials. One should vary *η* and *θ* to determine the sensitivity of the comparison to changes in the assumptions. It should be noted that it may be unrealistic to assume no bias in a clinical trial. However more experience through re-analysis of existing data is necessary to derive well-founded estimates for *η* and *θ*.

#### **Case Study (EnBand-Study)**

(continued). To choose an appropriate randomization procedure for the EnBand-Study, a comprehensive simulation study will be conducted to assess the impact of the choice of a randomization procedure on the type I error probability assuming selection and chronological bias. Considering the estimates *η*=0.09,*θ*=0.26 and *σ*=0.73 derived from the SPR-Study, we are interested in investigate the stability of our calculation with respect to rather small deviations from the estimates. Accordingly, we considered the “50% change” from estimates as values for our sensitivity analysis, resulting in the values 0.04,0.09 and 0.14 for *η* and 0.13,0.26 and 0.39 for *θ* in the sensitivity study. Of course, other arguments, e.g. using the limits of the 95% confidence interval of the estimates may be used as basis for the sensitivity analysis. However, it should be noted, that in our special case with the 95%-CI for *η* with (−0.09;0.26) and for *θ* with (−0.18;0.71) large deviations from the estimates on the on hand and large values compared to the expected effect size shown up, which be might a source of discussion.

### Step 4. Software

This paragraph should specify the software used for the evaluation. In general, the evaluation is not restricted to a specific software. However, to the best of our knowledge there is only one comprehensive software solution available for the assessment of randomization procedures. Recently Uschner [10] published the *R* package *randomizeR* which provides the basis for the computations for our case study. The package is available at the Comprehensive R Archive Network. It currently includes fifteen randomization procedures and allows the generation of randomization sequences in *R* and as a .csv file. Different assessment criteria, like selection and chronological bias and the combination of both are implemented in the software to assess and compare randomization procedures as well.

#### **Case Study (EnBand-Study)**

(continued). The randomizeR software version 1.3 will be used in the simulation study.

### Step 5. Results

The results calculated with *randomizeR* can be shown for a range of sequences as well as using summary statistics. Within this section, first some analytical examples are given followed by numerical results for the underlying example.

#### **Case Study (EnBand-Study)**

(continued). Using the settings above for the selection and chronological bias effects, the numerical results for the evaluation are given in Tables 2, 3 and 4. They were derived by Monte Carlo simulation with *r*=100000 randomization sequences. This ensures an accuracy with two decimal digits after the decimal point for *P*_*RP*_(*ω*≤0.05) and 3 decimal digits for the mean type I error probabilities. The sequences were generated according specific randomization procedures implemented in *randomizeR*. Using the type of bias as specified (selection bias, chronological bias, both types of bias) and the parameters (*η*, *θ*), the type I error probability is computed for each randomization sequence using the doubly non-central *t*-distribution with non-centrality parameters *δ* and *λ*. From these *r*=100000 type I error probabilities the mean value and the the probability of sequences exhibiting a type I error probability less than or equal to 0.05 are calculated for each randomization procedure.

**Table 2:** Impact of selection bias effect *η*=0.09 and linear time trend effect *θ*=0.26 with *σ*=0.73 on probability of type I error for different randomization procedures

Randomization	Type I Error	*P*_*RP*_(*ω*≤0.05)
Procedure	Probability	
[*R* *P*]	[mean]	
CR	0.050	0.53
RAR	0.052	0.34
PBR(2)	0.105	0.00
PBR(10)	0.069	0.00
BSD(3)	0.054	0.11
BSD(4)	0.052	0.34
BSD(5)	0.051	0.46
MP(3)	0.062	0.00
MP(4)	0.058	0.01
MP(5)	0.055	0.06
EBC(0.67)	0.062	0.02
CHEN(2, 0.67)	0.072	0.00
CHEN(3, 0.67)	0.066	0.00
CHEN(4, 0.67)	0.064	0.00
CHEN(5, 0.67)	0.063	0.01
UD(0,1)	0.051	0.44
UD(1,2)	0.051	0.46

**Table 3:** Impact of selection bias effect *η*=0.09 and linear time trend effect *θ*=0.26 with *σ*=0.73 on probability of type I error for the big stick design

Randomization	Type I Error	*P*_*RP*_(*ω*≤0.05)
Procedure	Probability	
[*R* *P*]	[mean]	
BSD(10)	0.050	0.53
BSD(15)	0.051	0.51
BSD(20)	0.050	0.52
BSD(25)	0.050	0.53
BSD(30)	0.050	0.53
BSD(35)	0.050	0.53
BSD(40)	0.050	0.52

**Table 4:** Impact of selection bias effect *η*=0.09 and linear time trend effect *θ*=0.26 with *σ*=0.73 on probability of type I error for Wei’s urn design

Randomization	Type I Error	*P*_*RP*_(*ω*≤0.05)
Procedure	Probability	
[*R* *P*]	[mean]	
UD(0,1)	0.051	0.44
UD(0,2)	0.051	0.44
UD(0,3)	0.051	0.44
UD(1,1)	0.051	0.47
UD(1,2)	0.051	0.46
UD(1,3)	0.051	0.45
UD(2,1)	0.051	0.48
UD(2,2)	0.051	0.47
UD(2,3)	0.051	0.46

The results in Table 2 show that complete randomization performs best in both criteria if both biases are present in the study data. We observed promising results for BSD and UD. Thus we investigated these two procedures in more detail with respect to their parameters, in particular because complete randomization is criticized due to the imbalance behavior. The performance of the big stick design and Wei’s urn design increases with respect to the probability of sequences exhibiting a type I error probability less than or equal to 0.05. Taking into account that a certain amount of imbalance does not affect the power of the test, we varied the maximum tolerated imbalance of the big stick design (see Table 3) and the parameters of Wei’s urn design (see Table 4) to get a more detailed image of the performance of these two design with respect to their parameters.

The data in Tables 3 and 4 show that the big stick design with *a*=10 performs reasonably well if selection bias as well as linear time trend are present. Of course, the joint biasing policy hides individual selection or time trend effects. The results in Tables 2, 3 and 4 are extended to individual selection and/or time trend effects in an additional file in more detail [see Additional file 1].

Table 5 shows the results of varying *η* and *θ* for these randomization procedures (complete randomization and the big stick design). Details for individual as well as joint selection bias and linear time trend effects, as well as for no effects and upper limit of 95% confidence intervals are included in an additional file [see Additional file 2].

**Table 5:** 50% change of selection bias effect and linear time trend effect (*η*,*θ*) on probability of type I error for complete randomization (CR) and big stick design (BSD)

Randomization	Selection	Linear-Time	Type I Error	*P*_*RP*_(*ω*≤0.05)
Procedure	Bias	Trend Bias	Probability	
[*R* *P*]	*η*	*θ*	[mean]	
CR	0.04	0.13	0.050	0.52
CR	0.09	0.26	0.050	0.53
CR	0.14	0.39	0.051	0.56
BSD(3)	0.04	0.13	0.051	0.10
BSD(3)	0.09	0.26	0.054	0.11
BSD(3)	0.14	0.39	0.059	0.10
BSD(4)	0.04	0.13	0.050	0.32
BSD(4)	0.09	0.26	0.052	0.34
BSD(4)	0.14	0.39	0.053	0.34
BSD(5)	0.04	0.13	0.050	0.45
BSD(5)	0.09	0.26	0.051	0.46
BSD(5)	0.14	0.39	0.051	0.47
BSD(10)	0.04	0.13	0.050	0.52
BSD(10)	0.09	0.26	0.050	0.53
BSD(10)	0.14	0.39	0.050	0.57

### Step 6. Discussion and Conclusion

This step concerns the discussion of the results and their interpretation with particular regard to the trial setting.

#### **Case Study (EnBand-Study)**

(continued). From Tables 2, 3 and 4 we see that, with the setting *η*=0.09 and *θ*=0.26, there are large differences between the performance of the randomization procedures. Even complete randomization does not prevent against selection and time trend bias overall; see first line of Table (2). It should be noted that the latter is already known from Rosenberger and Lachin [2]. As a matter of fact, using complete randomization, almost 50% of the randomization sequences exhibit a type I error probability elevation. Almost similar results can be observed for the BSD(5) and UD. Using the PBR with block sizes 2 or 10, the big stick design with maximal imbalance of *a*=3, MP(3,4,5), EBC(2/3), and Chen’s design are not recommended for application in our trial setting based on the probability of sequences exhibiting a type I error probability less than or equal to 0.05. Table 3 shows that the performance of the big stick design improves depending on the maximal tolerated imbalance up to 10. For Wei’s urn design, from Table 4, we see similar results over the parameters considered without reaching the big stick design level.

It is often misleading to focus on the “mean type *I* error probability” (see second column of Table 2) which shows comparable results for CR, RAR, BSD(5), UD(0,1) and UD(1,2). The variability can clearly be seen by looking at the probability of sequences exhibiting a type I error probability less than or equal to 0.05.

The above argument may imply that one should relax the terminal balance requirement by using the big stick design to achieve better results. Indeed, if the investigator is willing to accept imbalance in the data, say by 40 patients, it results an acceptable loss of power. However, in the case of BSD(10) 53% of allocation sequences under selection bias (0.09) and time trend (0.26) still preserve the type I error probability of 0.05. So the maximum tolerated imbalance can be restricted to 10 for BSD.

It should be taken into account that the evaluation above uses small selection and chronological bias effects in the example above. This may be different in other clinical settings. However, the evaluation shows that ignoring the influence of selection bias as well as chronological bias may affect the test decision by means of type I error rate probability. The effect may be conservative or anti-conservative test decisions.

#### **Case Study (EnBand-Study)**

We conclude that with a selection bias effect of *η*=0.09 and a linear time trend of 0.26*i*/*n*, the impact of the joint additive bias on the type I error probability inflation is kept to an acceptable minimum by complete randomization and the big stick design with maximum tolerated imbalance of 10. Although complete randomization performs slightly better in the case study, we will use the big stick design in the analysis of the EnBand-Study, because it controls also the maximum tolerated imbalance that may influence power calculations.

## Discussion

Randomization is considered to be the most important design feature in randomized clinical trials to protect against bias [3, 33]. However no scientifically-based recommendation or argument for selecting a randomization procedure has been given or proposed in the literature up to now.

In this paper, the ERDO template is proposed to give scientific arguments for the selection of a randomization procedure with respect to the clinical situation under investigation including a template for a structured report in the design phase of a clinical trial. A new evaluation metric based on the doubly non-central *t* distribution and a joint assessment criterion were derived as well. This enables a scientific evaluation of randomization procedures by using the randomizeR software comparably to what is commonly conducted for sample size or power considerations.

ERDO can be applied to every clinical trial, taking into account, that in every practical situation different forms and strengthen of selection biases and/or time trend bias could affect the test decision. Practical recommendations about the amount of selection bias and/or time trend effect may be derived from previous studies, or in case of uncertainty, as fraction from effect size and /or variation. The calculation can be easily conducted with our randomizeR software package [10]. Reporting a clinical trial in a medical journal requires a concise description of the selected randomization procedure and the scientific criteria on which that selection was based.

### Limitations

One limitation of our model for selection bias is, that it appears to be not reasonable if the population is very limited, so that there is a pressure to include every patient in the study. This happens in clinical trials with very rare diseases. Tamm et al. [8], considered cases with relaxed selection bias policies and misclassification, showing that selection bias does not completely vanish. In limited populations with high pressure to recruit, by means that every patient has to be enrolled, e.g. by an external enrollment board, it can be argued, that the effect of selection bias vanishes. We interpret our selection biasing policy as modeling the (worst case) related to unconscious selection of patients.

It has to be mentioned, that the less knowledge the designers have, the more appealing pure randomization or the procedures closest to pure randomization will be. Similarities between sample size justification and our investigation to justify the selection of the randomization procedure now imply a discussion about possible biases in clinical trials. In our special example the magnitude of the time trend seem to dominate the selection bias. This may alter in other clinical settings.

In general we would recommend to assume at least a certain amount of selection bias effect as well as time trend effect, which may go in line with the assumed effect size. And, taking into account open access to clinical data recommendation, there will be a gain in information in the future, which could be used in better designing a clinical trial.

### Extensions

We propose ERDO as a framework for “optimal” selection of a randomization procedure in clinical trials with regard to avoid bias. Hereby, we considered the most prominent types selection and linear time trend bias in randomized clinical trials. However, other types of bias can be easily implemented in ERDO, if a corresponding statistical model can be formulated. In addition, the ERDO approach can be applied to other metrics as well, see e.g. Schindler [30]. Some of them like averaged number of best guesses or loss in power are already implemented in randomizeR, which constitutes the computational basic. And finally other randomization procedures e.g. covariate adaptive randomization procedures can be included in the evaluation process. Although the current presentation is restricted to a two arm parallel group design, an extended biasing policy for multi-arm clinical trials with continuous endpoint was developed and briefly studied (Uschner et al.: The Impact of Selection Bias in Randomized Multi-Arm Parallel Group Clinical Trials, submitted). Similarly Rückbeil [9] proposed a biasing policy for survival data and conducted a comparison study. Both approaches will be implemented in the software in the upcoming release. We are working on extending the results to other endpoints and to group sequential clinical trials. The methods can already be used in stratified trials by analyzing the strata separately and pooling the results. Our presented results for the bias model in a single centre study can be translated to multicentre clinical trials in cases, where all centres follow the same biasing policy and the time trend is independent of the centre.

Another important extension concerns a selection bias corrected test [21] which has been introduced for a maximum likelihood based test. The bias corrected test is suitable if large sample arguments can be used to support the application of parametric tests. However, particularly in small samples where the asymptotic arguments are questionable, randomization based inference may be a suitable alternative.

Of course many possible metrics may be considered. If the particular evaluation criteria are measured on different scales, Schindler [30] proposed a uniform assessment criterion. Other metrics may also be used to combine criteria. In *randomizeR* a linked assessment criteria via Derringer Suich desirability [34] functions is implemented.

Second, concerning the balancing criteria, although it is known that power is maximized in balanced designs using continuous and homoscedastic endpoints, some slight deviation from balance occurs by enrollment and missing observation in almost every clinical trial. Moreover, the power of 80% is nearly kept in a study with an allocation ratio of 2:1 or 2:3 if the effect size of ranges between 0.2 and 0.8 in a two-sample *t*-test with two-sided hypothesis at significance level 5%. So randomization procedures which allow for moderate imbalances should not be excluded from consideration.

Further, varying *η* and *θ* can be used to illustrate their effects and bring sound scientific arguments into the choice of a randomization procedure. However, choices of appropriate *η* and *θ* in practice will also depend on the disease and study design restrictions (e.g., if masking is possible).

Connected to the randomization procedure is the correct choice of the statistical analysis. Some authors have argued that randomization-based inference is to be preferred in particular if the sample size is small. Of course the possible allocation sets resulting from the different randomization procedures might be taken into account for the selection.

## Conclusion

We acknowledge that until now the assessment of randomization procedures in clinical trials has been complex and difficult for investigators to accomplish. However, the methodology presented in this article facilitates this task.

The proposed ERDO framework guides the investigator through a template for the choice of a randomization procedure, and provides easy to use tools for the assessment. The barriers for the thorough reporting and assessment of randomization procedures could be further reduced in the future when regulators and pharmaceutical companies employ similar, standardized frameworks for the choice of randomization procedures.

## Additional files

Additional file 1 This document includes detailed tables of the impact of selection bias effect *η* and linear time trend effect *θ* separately and jointly on the type I error probability for different randomization procedures. (PDF 49 kb)

Additional file 2 This document includes detailed tables of the sensitivity analysis. The amount of *η* and *θ* is varied from the estimate by “50% changes”, resulting in the values 0.04, 0.09 and 0.14 for *η*and 0.13, 0.26 and 0.39 for *θ*. Further the upper limit of the 95%-CI for *η*with (-0.09; 0.26) and for *θ*with (-0.18; 0.71) are used as well as the point of no bias *η*=0, *θ*=0. All resulting combinations of *η* and *θ* are considered for the different randomization procedures. (PDF 74 kb)

## Abbreviations

ANOVA: Analysis of variance
ANCOVA: Analysis of covariance
BSD(*a*): Big stick design with maximal tolerated imbalance *a*
Chen(*a*,*p*): Chen’s design with a maximum tolerated imbalance *a*
CONSORT: Consolidated standards of reporting trials
CR: Complete randomization
EBC(*p*): Efron’s biased coin with probability *p*
EnBand-Study: encircling band study
ERDO: Evaluation of randomization procedures for design optimization
ICH: International committee of harmonization
MAR: Minimal angle of resolution
MP(*a*): Maximal procedure with a maximum tolerated imbalance *a*
*P*_*RP*_(*ω*≤0.05): Probability of sequences of a randomization procedure *RP* exhibiting a type I error probability *q* less than or equal to 0.05
PBR(b): Permuted block randomization with block size *b*
PPV: Pars Plana vitrectomy
RAR: Random allocation rule
SB: Scleral buckling
SPR-Study: Scleral buckling versus pars plana vitrectomy study
SD: Standard deviation
UD(*α*,*β*): Wei’s urn design

## References

[1] Senn S (2004). Controversies concerning randomization and additivity in clinical trials. Stat Med.

[2] Rosenberger WF, Lachin J (2002). Randomization in Clinical Trials: Theory and Practice.

[3] ICH E9. Statistical principles for clinical trials. 1998. http://www.ich.org/fileadmin/Public_Web_Site/ICH_Products/Guidelines/Efficacy/E9/Step4/E9_Guideline.pdf. Access 30 Aug 2016.

[4] Schulz KF, Altman DG, Moher D, for the CONSORT Group (2010). CONSORT 2010 Statement: updated guidelines for reporting parallel group randomised trials. PLoS Med.

[5] Kahan BC, Morris TP (2012). Reporting and analysis of trials using stratified randomisation in leading medical journals: review and reanalysis. BMJ.

[6] Proschan M (1994). Influence of selection bias on type I error rate under random permuted block designs. Stat Sin.

[7] Kennes LN, Cramer E, Hilgers RD, Heussen N (2011). The impact of selection bias on test decisions in randomized clinical trials. Stat Med.

[8] Tamm M, Cramer E, Kennes LN, Heussen N (2012). Influence of Selection Bias on the Test Decision - A Simulation Study. Methods Inf Med.

[9] Rückbeil MV, Hilgers RD, Heussen N (2011). Assessing the impact of selection bias on test decisions in trials with time-to-event outcome. Stat Med.

[10] Uschner D, Schindler D, Hilgers RD, Heussen N. randomizeR: An R Package for the Assessment and Implementation of Randomization in Clinical Trials. J Stat Softw. 2017. http://www.ideal.rwth-aachen.de/wp-content/uploads/2017/11/article_accepted.pdf.

[11] Blackwell D, Hodges J (1957). Design for the control of selection bias. Ann Math Stat.

[12] Berger VW (2005). Selection Bias and Covariate Imbalances in randomized Clinical Trials.

[13] Joppi R, Garattini S, Bertele’ V (2013). Orphan drugs, orphan diseases. The first decade of orphan drug legislation in the EU. Eur J Clin Pharmacol.

[14] Altman DG, Royston JP (1988). The hidden effect of time. Methods Inf Med.

[15] Tamm M, Hilgers RD (2014). Chronological Bias in Randomized Clinical Trials Arising from Different Types of Unobserved Time Trends. Methods Inf Med.

[16] Benda N, Branson M, Maurer W, Friede T (2010). Aspects of Modernizing Drug Development Using Clinical Scenario Planning and Evaluation. Drug Inf J.

[17] Heinrich H, Bartz-Schmidt KU, Bornfeld N, Weiss C, Hilgers RD, Foerster MH (2007). Scleral Buckling versus Primary Vitrectomy in Rhegmatogenous Retinal Detachment Study Group. Scleral Buckling versus Primary Vitrectomy in Rhegmatogenous Retinal Detachment : A Prospective Randomized Multicenter Clinical Study Ophthalmology.

[18] Hopper AN, Jamison MH, Le WG (2007). Learning curves in surgical practice. Postgrad Med J.

[19] Berger VW, Grant WC, Vazquez LF (2010). Sensitivity designs for preventing bias replication in randomized clinical trials. Stat Methods Med Res.

[20] Koenig F, Slattery J, Groves T, Lang T, Benjamini Y, Day S, Bauer P, Posch M (2014). Sharing clinical trial data on patient level: Opportunities and challenges. Biom J.

[21] Kennes LN, Rosenberger WF, Hilgers RD (2015). Inference for blocked randomization under a selection bias model. Biometrics.

[22] Efron B (1971). Forcing a sequential experiment to be balanced. Biometrika.

[23] Berger VW, Ivanova A, Knoll DM (2003). Minimizing Predictability while Retaining Balance through the Use of Less Restrictive Randomization Procedures. Stat Med.

[24] Wei LJ (1977). A Class of Designs for Sequential Clinical Trials. J Am Stat Assoc.

[25] Soares JF, Wu CFJ (1982). Some restricted randomization rules in sequential designs. Commun Stat Theory Methods.

[26] Chen YP (1999). Biased coin design with imbalance tolerance. Commun Stat Stoch Model.

[27] Antognini AB, Giovagnoli A (2004). New ’Biased Coin Design’ for the Sequential Allocation of Two Treatments. J R Stat Soc C.

[28] Berger VW, Bejleri K, Agnor R (2016). Comparing MTI Randomization Procedures to Blocked Randomization. Stat Med.

[29] Atkinson A (2003). Minimizing predictability while retaining balance through the use of less restrictive randomization procedures. Stat Med.

[30] Schindler D. Assessment of Randomization Procedures in the Presence of Selection and Chronological Bias. PhD Thesis, University of Dortmund. Germany; 2016.

[31] Langer S. The modified distribution of the t-test statistic under the influence of selection bias based on random allocation rule. Master Thesis, RWTH Aachen University. Germany; 2014.

[32] Balakrishnan N, Kotz S, Johnson NL (1995). Continuous univariate distributions Volume 2.

[33] Schulz KF, Altman DG, Moher D, for the CONSORTGroup (2010). CONSORT 2010 Statement: updated guidelines for reporting parallel group randomised trials. PLoS Med.

[34] Derringer GC, Suich R (1980). Simultaneous optimization of several response variables. J Qual Technol.

