# Supplementary material for: ERDO - a framework to select an appropriate randomization procedure for clinical trials
Source: BMC Med Res Methodol. 2017 Dec 4;17:159. doi: 10.1186/s12874-017-0428-z (PMC5715815; doi:10.1186/s12874-017-0428-z)
Supplement: Supplementary file 1 — This document includes detailed tables of the impact of selection bias effect η and linear time trend effect θ separately and jointly on the type I error probability for different randomization procedures. (PDF 49 kb) [file 12874_2017_428_MOESM1_ESM.pdf]

This document includes detailed tables of the impact of selection bias effect η and linear time trend effect θ separately and jointly on the type I error probability for different randomization procedures.

Table 1: Impact of selection bias effect $\eta = 0.09$ and linear time trend effect $\theta = 0.26$ with $\sigma = 0.73$ on probability of type I error for different randomization procedures

Randomization Procedure [RP]	Type I Error Probability [mean]	$P_{RP}(\omega \leq 0.05)$
CR	0.050	0.53
RAR	0.052	0.34
PBR(2)	0.105	0.00
PBR(10)	0.069	0.00
BSD(3)	0.054	0.11
BSD(4)	0.052	0.34
BSD(5)	0.051	0.46
MP(3)	0.062	0.00
MP(4)	0.058	0.01
MP(5)	0.055	0.06
EBC(0.67)	0.062	0.02
CHEN(2, 0.67)	0.072	0.00
CHEN(3, 0.67)	0.066	0.00
CHEN(4, 0.67)	0.064	0.00
CHEN(5, 0.67)	0.063	0.01
UD(0,1)	0.051	0.44
UD(1,2)	0.051	0.46

Table 2: Impact of selection bias effect $\eta = 0.09$ with $\sigma = 0.73$ on probability of type I error for different randomization procedures

Randomization Procedure [RP]	Type I Error Probability [mean]	$P_{RP}(\omega \leq 0.05)$
CR	0.050	0.47
RAR	0.052	0.12
PBR(2)	0.107	0.00
PBR(10)	0.071	0.00
BSD(3)	0.055	0.00
BSD(4)	0.053	0.10
BSD(5)	0.052	0.23
MP(3)	0.064	0.00
MP(4)	0.059	0.00
MP(5)	0.056	0.00
EBC(0.67)	0.064	0.00
CHEN(2, 0.67)	0.074	0.00
CHEN(3, 0.67)	0.068	0.00
CHEN(4, 0.67)	0.065	0.00
CHEN(5, 0.67)	0.064	0.00
UD(0,1)	0.052	0.23
UD(1,2)	0.052	0.26

Table 3: Impact of linear time trend effect $\theta = 0.26$ with $\sigma = 0.73$ on probability of type I error for different randomization procedures

Randomization Procedure $[RP]$	Type I Error Probability [mean]	$P_{RP}(\omega \leq 0.05)$
CR	0.050	0.67
RAR	0.050	0.66
PBR(2)	0.049	1.00
PBR(10)	0.049	1.00
BSD(3)	0.049	1.00
BSD(4)	0.049	0.99
BSD(5)	0.049	0.95
MP(3)	0.049	1.00
MP(4)	0.049	1.00
MP(5)	0.049	1.00
EBC(0.67)	0.049	0.99
CHEN(2, 0.67)	0.049	1.00
CHEN(3, 0.67)	0.049	1.00
CHEN(4, 0.67)	0.049	1.00
CHEN(5, 0.67)	0.049	1.00
UD(0,1)	0.050	0.81
UD(1,2)	0.050	0.81

Table 4: Impact of selection bias effect $\eta = 0.09$ and linear time trend effect $\theta = 0.26$ with $\sigma = 0.73$ on probability of type I error for Big Stick with varying MTI

Randomization Procedure $[RP]$	Type I Error Probability [mean]	$P_{RP}(\omega \leq 0.05)$
BSD(10)	0.050	0.53
BSD(15)	0.051	0.51
BSD(20)	0.050	0.52
BSD(25)	0.050	0.53
BSD(30)	0.050	0.53
BSD(35)	0.050	0.53
BSD(40)	0.050	0.52

Table 5: Impact of selection bias effect $\eta = 0.09$ with $\sigma = 0.73$ on probability of type I error for Big Stick with varying MTI

Randomization Procedure $[RP]$	Type I Error Probability [mean]	$P_{RP}(\omega \leq 0.05)$
BSD(10)	0.051	0.41
BSD(15)	0.050	0.45
BSD(20)	0.050	0.46
BSD(25)	0.050	0.47
BSD(30)	0.050	0.47
BSD(35)	0.050	0.47
BSD(40)	0.050	0.47

Table 6: Impact of linear time trend effect $\theta = 0.26$ with $\sigma = 0.73$ on probability of type I error for Big Stick with varying MTI

Randomization Procedure [RP]	Type I Error Probability [mean]	$P_{RP}(\omega \leq 0.05)$
BSD(10)	0.050	0.69
BSD(15)	0.050	0.65
BSD(20)	0.050	0.66
BSD(25)	0.050	0.66
BSD(30)	0.050	0.67
BSD(35)	0.050	0.67
BSD(40)	0.050	0.67

Table 7: Impact of selection bias effect $\eta = 0.09$ and linear time trend effect $\theta = 0.26$ with $\sigma = 0.73$ on probability of type I error for various Wei's Urn Designs

Randomization Procedure [RP]	Type I Error Probability [mean]	$P_{RP}(\omega \leq 0.05)$
UD(0,1)	0.051	0.44
UD(0,2)	0.051	0.44
UD(0,3)	0.051	0.44
UD(1,1)	0.051	0.47
UD(1,2)	0.051	0.46
UD(1,3)	0.051	0.45
UD(2,1)	0.051	0.48
UD(2,2)	0.051	0.47
UD(2,3)	0.051	0.46

Table 8: Impact of selection bias effect $\eta = 0.09$ and with $\sigma = 0.73$ on probability of type I error for various Wei's Urn Designs

Randomization Procedure [RP]	Type I Error Probability [mean]	$P_{RP}(\omega \leq 0.05)$
UD(0,1)	0.052	0.23
UD(0,2)	0.052	0.24
UD(0,3)	0.052	0.23
UD(1,1)	0.051	0.28
UD(1,2)	0.052	0.26
UD(1,3)	0.052	0.26
UD(2,1)	0.051	0.30
UD(2,2)	0.051	0.28
UD(2,3)	0.051	0.27

Table 9: Impact of linear time trend effect $\theta = 0.26$ with $\sigma = 0.73$ on probability of type I error for various Wei's Urn Designs

Randomization Procedure [RP]	Type I Error Probability [mean]	$P_{RP}(\omega \leq 0.05)$
UD(0,1)	0.050	0.81
UD(0,2)	0.050	0.81
UD(0,3)	0.050	0.81
UD(1,1)	0.050	0.80
UD(1,2)	0.050	0.81
UD(1,3)	0.050	0.80
UD(2,1)	0.050	0.79
UD(2,2)	0.050	0.80
UD(2,3)	0.050	0.80
