# Supplementary material for: ERDO - a framework to select an appropriate randomization procedure for clinical trials
Source: BMC Med Res Methodol. 2017 Dec 4;17:159. doi: 10.1186/s12874-017-0428-z (PMC5715815; doi:10.1186/s12874-017-0428-z)
Supplement: Supplementary file 2 — This document includes detailed tables of the sensitivity analysis. The amount of η and θ is varied from the estimate by “50% changes”, resulting in the values 0.04, 0.09 and 0.14 for ηand 0.13, 0.26 and 0.39 for θ. Further the upper limit of the 95%-CI for ηwith (-0.09; 0.26) and for θwith (-0.18; 0.71) are used as well as the point of no bias η=0, θ=0. All resulting combinations of η and θ are considered for the different randomization procedures. (PDF 74 kb) [file 12874_2017_428_MOESM2_ESM.pdf]

This document includes detailed tables of the sensitivity analysis. The amount of η and θ is varied from the estimate by "50% changes", resulting in the values $0.04, 0.09$ and 0.14 for η and $0.13, 0.26$ and 0.39 for θ. Further the upper limit of the 95%-CI for η with $(-0.09; 0.26)$ and for θ with $(-0.18; 0.71)$ are used as well as the point of no bias $\eta = 0, \theta = 0$. All resulting combinations of η and θ are considered for the different randomization procedures.

Table 1: Sensitivity analysis for CR

Randomization Procedure [RP]	Selection Bias Effect [η]	Linear-Time Trend Bias Effect [θ]	Type I Error Probability [mean]	$P_{RP}(\omega \leq 0.05)$
CR	0.00	0.00	0.050	0.00
CR	0.00	0.13	0.050	0.66
CR	0.00	0.26	0.050	0.67
CR	0.00	0.39	0.050	0.67
CR	0.00	0.71	0.050	0.69
CR	0.04	0.00	0.050	0.42
CR	0.04	0.13	0.050	0.52
CR	0.04	0.26	0.050	0.58
CR	0.04	0.39	0.050	0.64
CR	0.04	0.71	0.050	0.68
CR	0.09	0.00	0.050	0.47
CR	0.09	0.13	0.050	0.51
CR	0.09	0.26	0.050	0.53
CR	0.09	0.39	0.050	0.59
CR	0.09	0.71	0.050	0.65
CR	0.14	0.00	0.051	0.51
CR	0.14	0.13	0.051	0.52
CR	0.14	0.26	0.051	0.52
CR	0.14	0.39	0.051	0.56
CR	0.14	0.71	0.051	0.61
CR	0.26	0.00	0.052	0.46
CR	0.26	0.13	0.052	0.52
CR	0.26	0.26	0.052	0.55
CR	0.26	0.39	0.052	0.55
CR	0.26	0.71	0.052	0.58

Table 2: Sensitivity analysis for RAR

Randomization Procedure [RP]	Selection Bias Effect [η]	Linear-Time Trend Bias Effect [θ]	Type I Error Probability [mean]	$P_{RP}(\omega \leq 0.05)$
RAR	0.00	0.00	0.050	0.00
RAR	0.00	0.13	0.050	0.65
RAR	0.00	0.26	0.050	0.66
RAR	0.00	0.39	0.050	0.67
RAR	0.00	0.71	0.050	0.68
RAR	0.04	0.00	0.050	0.14
RAR	0.04	0.13	0.050	0.33
RAR	0.04	0.26	0.051	0.47
RAR	0.04	0.39	0.050	0.58
RAR	0.04	0.71	0.050	0.66
RAR	0.09	0.00	0.052	0.12
RAR	0.09	0.13	0.052	0.25
RAR	0.09	0.26	0.052	0.34
RAR	0.09	0.39	0.052	0.43
RAR	0.09	0.71	0.052	0.56
RAR	0.14	0.00	0.055	0.15
RAR	0.14	0.13	0.055	0.23
RAR	0.14	0.26	0.055	0.30
RAR	0.14	0.39	0.055	0.35
RAR	0.14	0.71	0.054	0.47
RAR	0.26	0.00	0.065	0.17
RAR	0.26	0.13	0.065	0.21
RAR	0.26	0.26	0.065	0.25
RAR	0.26	0.39	0.065	0.29
RAR	0.26	0.71	0.064	0.36

Table 3: Sensitivity analysis for PBR(2)

Randomization Procedure [RP]	Selection Bias Effect [η]	Linear-Time Trend Bias Effect [θ]	Type I Error Probability [mean]	$P_{RP}(\omega \leq 0.05)$
PBR(2)	0.00	0.00	0.050	0.00
PBR(2)	0.00	0.13	0.050	1.00
PBR(2)	0.00	0.26	0.049	1.00
PBR(2)	0.00	0.39	0.047	1.00
PBR(2)	0.00	0.71	0.042	1.00
PBR(2)	0.04	0.00	0.061	0.00
PBR(2)	0.04	0.13	0.061	0.00
PBR(2)	0.04	0.26	0.060	0.00
PBR(2)	0.04	0.39	0.058	0.00
PBR(2)	0.04	0.71	0.052	0.00
PBR(2)	0.09	0.00	0.107	0.00
PBR(2)	0.09	0.13	0.106	0.00
PBR(2)	0.09	0.26	0.105	0.00
PBR(2)	0.09	0.39	0.102	0.00
PBR(2)	0.09	0.71	0.093	0.00
PBR(2)	0.14	0.00	0.190	0.00
PBR(2)	0.14	0.13	0.189	0.00
PBR(2)	0.14	0.26	0.187	0.00
PBR(2)	0.14	0.39	0.183	0.00
PBR(2)	0.14	0.71	0.169	0.00
PBR(2)	0.26	0.00	0.510	0.00
PBR(2)	0.26	0.13	0.509	0.00
PBR(2)	0.26	0.26	0.506	0.00
PBR(2)	0.26	0.39	0.501	0.00
PBR(2)	0.26	0.71	0.480	0.00

Table 4: Sensitivity analysis for PBR(10)

Randomization Procedure [RP]	Selection Bias Effect [η]	Linear-Time Trend Bias Effect [θ]	Type I Error Probability [mean]	$P_{RP}(\omega \leq 0.05)$
PBR(10)	0.00	0.00	0.050	0.00
PBR(10)	0.00	0.13	0.050	1.00
PBR(10)	0.00	0.26	0.049	1.00
PBR(10)	0.00	0.39	0.047	1.00
PBR(10)	0.00	0.71	0.042	1.00
PBR(10)	0.04	0.00	0.054	0.00
PBR(10)	0.04	0.13	0.054	0.00
PBR(10)	0.04	0.26	0.053	0.00
PBR(10)	0.04	0.39	0.051	0.10
PBR(10)	0.04	0.71	0.045	1.00
PBR(10)	0.09	0.00	0.071	0.00
PBR(10)	0.09	0.13	0.070	0.00
PBR(10)	0.09	0.26	0.069	0.00
PBR(10)	0.09	0.39	0.067	0.00
PBR(10)	0.09	0.71	0.060	0.01
PBR(10)	0.14	0.00	0.099	0.00
PBR(10)	0.14	0.13	0.098	0.00
PBR(10)	0.14	0.26	0.097	0.00
PBR(10)	0.14	0.39	0.095	0.00
PBR(10)	0.14	0.71	0.086	0.00
PBR(10)	0.26	0.00	0.216	0.00
PBR(10)	0.26	0.13	0.215	0.00
PBR(10)	0.26	0.26	0.213	0.00
PBR(10)	0.26	0.39	0.209	0.00
PBR(10)	0.26	0.71	0.195	0.00

Table 5: Sensitivity analysis for BSD(3)

Randomization Procedure [RP]	Selection Bias Effect [η]	Linear-Time Trend Bias Effect [θ]	Type I Error Probability [mean]	$P_{RP}(\omega \leq 0.05)$
BSD(3)	0.00	0.00	0.050	0.00
BSD(3)	0.00	0.13	0.050	1.00
BSD(3)	0.00	0.26	0.049	1.00
BSD(3)	0.00	0.39	0.048	1.00
BSD(3)	0.00	0.71	0.042	1.00
BSD(3)	0.04	0.00	0.051	0.01
BSD(3)	0.04	0.13	0.051	0.10
BSD(3)	0.04	0.26	0.050	0.48
BSD(3)	0.04	0.39	0.049	0.85
BSD(3)	0.04	0.71	0.043	1.00
BSD(3)	0.09	0.00	0.055	0.00
BSD(3)	0.09	0.13	0.055	0.02
BSD(3)	0.09	0.26	0.054	0.11
BSD(3)	0.09	0.39	0.052	0.28
BSD(3)	0.09	0.71	0.047	0.73
BSD(3)	0.14	0.00	0.062	0.01
BSD(3)	0.14	0.13	0.062	0.02
BSD(3)	0.14	0.26	0.061	0.04
BSD(3)	0.14	0.39	0.059	0.10
BSD(3)	0.14	0.71	0.053	0.39
BSD(3)	0.26	0.00	0.089	0.01
BSD(3)	0.26	0.13	0.089	0.01
BSD(3)	0.26	0.26	0.087	0.02
BSD(3)	0.26	0.39	0.086	0.03
BSD(3)	0.26	0.71	0.079	0.11

Table 6: Sensitivity analysis for BSD(4)

Randomization Procedure [RP]	Selection Bias Effect [η]	Linear-Time Trend Bias Effect [θ]	Type I Error Probability [mean]	$P_{RP}(\omega \leq 0.05)$
BSD(4)	0.00	0.00	0.050	0.00
BSD(4)	0.00	0.13	0.050	0.98
BSD(4)	0.00	0.26	0.049	0.99
BSD(4)	0.00	0.39	0.048	0.99
BSD(4)	0.00	0.71	0.043	0.99
BSD(4)	0.04	0.00	0.051	0.12
BSD(4)	0.04	0.13	0.050	0.32
BSD(4)	0.04	0.26	0.050	0.65
BSD(4)	0.04	0.39	0.048	0.86
BSD(4)	0.04	0.71	0.044	0.96
BSD(4)	0.09	0.00	0.053	0.10
BSD(4)	0.09	0.13	0.052	0.18
BSD(4)	0.09	0.26	0.052	0.34
BSD(4)	0.09	0.39	0.050	0.52
BSD(4)	0.09	0.71	0.045	0.80
BSD(4)	0.14	0.00	0.056	0.13
BSD(4)	0.14	0.13	0.056	0.16
BSD(4)	0.14	0.26	0.055	0.24
BSD(4)	0.14	0.39	0.053	0.34
BSD(4)	0.14	0.71	0.049	0.61
BSD(4)	0.26	0.00	0.068	0.15
BSD(4)	0.26	0.13	0.068	0.15
BSD(4)	0.26	0.26	0.067	0.18
BSD(4)	0.26	0.39	0.066	0.22
BSD(4)	0.26	0.71	0.061	0.35

Table 7: Sensitivity analysis for BSD(5)

Randomization Procedure [RP]	Selection Bias Effect [η]	Linear-Time Trend Bias Effect [θ]	Type I Error Probability [mean]	$P_{RP}(\omega \leq 0.05)$
BSD(5)	0.00	0.00	0.050	0.00
BSD(5)	0.00	0.13	0.050	0.94
BSD(5)	0.00	0.26	0.049	0.95
BSD(5)	0.00	0.39	0.048	0.94
BSD(5)	0.00	0.71	0.044	0.96
BSD(5)	0.04	0.00	0.050	0.25
BSD(5)	0.04	0.13	0.050	0.45
BSD(5)	0.04	0.26	0.050	0.69
BSD(5)	0.04	0.39	0.048	0.84
BSD(5)	0.04	0.71	0.044	0.93
BSD(5)	0.09	0.00	0.052	0.23
BSD(5)	0.09	0.13	0.051	0.31
BSD(5)	0.09	0.26	0.051	0.46
BSD(5)	0.09	0.39	0.049	0.61
BSD(5)	0.09	0.71	0.045	0.81
BSD(5)	0.14	0.00	0.053	0.28
BSD(5)	0.14	0.13	0.053	0.31
BSD(5)	0.14	0.26	0.052	0.38
BSD(5)	0.14	0.39	0.051	0.47
BSD(5)	0.14	0.71	0.047	0.67
BSD(5)	0.26	0.00	0.060	0.29
BSD(5)	0.26	0.13	0.060	0.30
BSD(5)	0.26	0.26	0.059	0.33
BSD(5)	0.26	0.39	0.059	0.36
BSD(5)	0.26	0.71	0.054	0.48

Table 8: Sensitivity analysis for BSD(10)

Randomization Procedure [RP]	Selection Bias Effect [η]	Linear-Time Trend Bias Effect [θ]	Type I Error Probability [mean]	$P_{RP}(\omega \leq 0.05)$
BSD(10)	0.00	0.00	0.050	0.00
BSD(10)	0.00	0.13	0.050	0.69
BSD(10)	0.00	0.26	0.050	0.69
BSD(10)	0.00	0.39	0.050	0.69
BSD(10)	0.00	0.71	0.049	0.72
BSD(10)	0.04	0.00	0.050	0.38
BSD(10)	0.04	0.13	0.050	0.52
BSD(10)	0.04	0.26	0.050	0.60
BSD(10)	0.04	0.39	0.050	0.67
BSD(10)	0.04	0.71	0.049	0.71
BSD(10)	0.09	0.00	0.051	0.41
BSD(10)	0.09	0.13	0.050	0.48
BSD(10)	0.09	0.26	0.050	0.53
BSD(10)	0.09	0.39	0.050	0.60
BSD(10)	0.09	0.71	0.049	0.67
BSD(10)	0.14	0.00	0.051	0.45
BSD(10)	0.14	0.13	0.051	0.49
BSD(10)	0.14	0.26	0.051	0.52
BSD(10)	0.14	0.39	0.050	0.57
BSD(10)	0.14	0.71	0.050	0.63
BSD(10)	0.26	0.00	0.053	0.43
BSD(10)	0.26	0.13	0.053	0.48
BSD(10)	0.26	0.26	0.053	0.52
BSD(10)	0.26	0.39	0.053	0.54
BSD(10)	0.26	0.71	0.052	0.58

Table 9: Sensitivity analysis for BSD(15)

Randomization Procedure [RP]	Selection Bias Effect [η]	Linear-Time Trend Bias Effect [θ]	Type I Error Probability [mean]	$P_{RP}(\omega \leq 0.05)$
BSD(15)	0.00	0.00	0.050	0.00
BSD(15)	0.00	0.13	0.050	0.64
BSD(15)	0.00	0.26	0.050	0.65
BSD(15)	0.00	0.39	0.050	0.65
BSD(15)	0.00	0.71	0.050	0.67
BSD(15)	0.04	0.00	0.050	0.40
BSD(15)	0.04	0.13	0.050	0.50
BSD(15)	0.04	0.26	0.050	0.55
BSD(15)	0.04	0.39	0.050	0.63
BSD(15)	0.04	0.71	0.050	0.66
BSD(15)	0.09	0.00	0.050	0.45
BSD(15)	0.09	0.13	0.050	0.49
BSD(15)	0.09	0.26	0.051	0.51
BSD(15)	0.09	0.39	0.050	0.57
BSD(15)	0.09	0.71	0.051	0.63
BSD(15)	0.14	0.00	0.051	0.49
BSD(15)	0.14	0.13	0.051	0.50
BSD(15)	0.14	0.26	0.051	0.50
BSD(15)	0.14	0.39	0.051	0.54
BSD(15)	0.14	0.71	0.051	0.60
BSD(15)	0.26	0.00	0.052	0.44
BSD(15)	0.26	0.13	0.052	0.50
BSD(15)	0.26	0.26	0.052	0.53
BSD(15)	0.26	0.39	0.052	0.53
BSD(15)	0.26	0.71	0.053	0.56

Table 10: Sensitivity analysis for BSD(20)

Randomization Procedure [RP]	Selection Bias Effect [η]	Linear-Time Trend Bias Effect [θ]	Type I Error Probability [mean]	$P_{RP}(\omega \leq 0.05)$
BSD(20)	0.00	0.00	0.050	0.00
BSD(20)	0.00	0.13	0.050	0.65
BSD(20)	0.00	0.26	0.050	0.66
BSD(20)	0.00	0.39	0.050	0.65
BSD(20)	0.00	0.71	0.050	0.67
BSD(20)	0.04	0.00	0.050	0.41
BSD(20)	0.04	0.13	0.050	0.50
BSD(20)	0.04	0.26	0.050	0.56
BSD(20)	0.04	0.39	0.050	0.63
BSD(20)	0.04	0.71	0.050	0.66
BSD(20)	0.09	0.00	0.050	0.46
BSD(20)	0.09	0.13	0.050	0.50
BSD(20)	0.09	0.26	0.050	0.52
BSD(20)	0.09	0.39	0.050	0.58
BSD(20)	0.09	0.71	0.051	0.63
BSD(20)	0.14	0.00	0.051	0.50
BSD(20)	0.14	0.13	0.051	0.51
BSD(20)	0.14	0.26	0.051	0.51
BSD(20)	0.14	0.39	0.051	0.55
BSD(20)	0.14	0.71	0.051	0.60
BSD(20)	0.26	0.00	0.052	0.45
BSD(20)	0.26	0.13	0.052	0.51
BSD(20)	0.26	0.26	0.052	0.53
BSD(20)	0.26	0.39	0.052	0.55
BSD(20)	0.26	0.71	0.053	0.56

Table 11: Sensitivity analysis for BSD(25)

Randomization Procedure [RP]	Selection Bias Effect [η]	Linear-Time Trend Bias Effect [θ]	Type I Error Probability [mean]	$P_{RP}(\omega \leq 0.05)$
BSD(25)	0.00	0.00	0.050	0.00
BSD(25)	0.00	0.13	0.050	0.65
BSD(25)	0.00	0.26	0.050	0.66
BSD(25)	0.00	0.39	0.050	0.66
BSD(25)	0.00	0.71	0.050	0.68
BSD(25)	0.04	0.00	0.050	0.41
BSD(25)	0.04	0.13	0.050	0.51
BSD(25)	0.04	0.26	0.050	0.57
BSD(25)	0.04	0.39	0.050	0.64
BSD(25)	0.04	0.71	0.050	0.67
BSD(25)	0.09	0.00	0.050	0.47
BSD(25)	0.09	0.13	0.050	0.50
BSD(25)	0.09	0.26	0.050	0.53
BSD(25)	0.09	0.39	0.050	0.58
BSD(25)	0.09	0.71	0.051	0.64
BSD(25)	0.14	0.00	0.051	0.51
BSD(25)	0.14	0.13	0.051	0.51
BSD(25)	0.14	0.26	0.051	0.52
BSD(25)	0.14	0.39	0.051	0.56
BSD(25)	0.14	0.71	0.051	0.61
BSD(25)	0.26	0.00	0.052	0.45
BSD(25)	0.26	0.13	0.052	0.52
BSD(25)	0.26	0.26	0.052	0.54
BSD(25)	0.26	0.39	0.052	0.55
BSD(25)	0.26	0.71	0.053	0.57

Table 12: Sensitivity analysis for BSD(30)

Randomization Procedure [RP]	Selection Bias Effect [η]	Linear-Time Trend Bias Effect [θ]	Type I Error Probability [mean]	$P_{RP}(\omega \leq 0.05)$
BSD(30)	0.00	0.00	0.050	0.00
BSD(30)	0.00	0.13	0.050	0.66
BSD(30)	0.00	0.26	0.050	0.67
BSD(30)	0.00	0.39	0.050	0.66
BSD(30)	0.00	0.71	0.050	0.68
BSD(30)	0.04	0.00	0.050	0.41
BSD(30)	0.04	0.13	0.050	0.52
BSD(30)	0.04	0.26	0.050	0.58
BSD(30)	0.04	0.39	0.050	0.64
BSD(30)	0.04	0.71	0.050	0.67
BSD(30)	0.09	0.00	0.050	0.47
BSD(30)	0.09	0.13	0.050	0.50
BSD(30)	0.09	0.26	0.050	0.53
BSD(30)	0.09	0.39	0.050	0.59
BSD(30)	0.09	0.71	0.050	0.64
BSD(30)	0.14	0.00	0.051	0.51
BSD(30)	0.14	0.13	0.051	0.52
BSD(30)	0.14	0.26	0.051	0.52
BSD(30)	0.14	0.39	0.051	0.56
BSD(30)	0.14	0.71	0.051	0.61
BSD(30)	0.26	0.00	0.052	0.46
BSD(30)	0.26	0.13	0.052	0.52
BSD(30)	0.26	0.26	0.052	0.54
BSD(30)	0.26	0.39	0.052	0.56
BSD(30)	0.26	0.71	0.053	0.57

Table 13: Sensitivity analysis for BSD(35)

Randomization Procedure [RP]	Selection Bias Effect [η]	Linear-Time Trend Bias Effect [θ]	Type I Error Probability [mean]	$P_{RP}(\omega \leq 0.05)$
BSD(35)	0.00	0.00	0.050	0.00
BSD(35)	0.00	0.13	0.050	0.66
BSD(35)	0.00	0.26	0.050	0.67
BSD(35)	0.00	0.39	0.050	0.66
BSD(35)	0.00	0.71	0.050	0.68
BSD(35)	0.04	0.00	0.050	0.41
BSD(35)	0.04	0.13	0.050	0.52
BSD(35)	0.04	0.26	0.050	0.58
BSD(35)	0.04	0.39	0.050	0.65
BSD(35)	0.04	0.71	0.050	0.68
BSD(35)	0.09	0.00	0.050	0.47
BSD(35)	0.09	0.13	0.050	0.51
BSD(35)	0.09	0.26	0.050	0.53
BSD(35)	0.09	0.39	0.050	0.59
BSD(35)	0.09	0.71	0.050	0.64
BSD(35)	0.14	0.00	0.051	0.50
BSD(35)	0.14	0.13	0.051	0.52
BSD(35)	0.14	0.26	0.051	0.52
BSD(35)	0.14	0.39	0.051	0.56
BSD(35)	0.14	0.71	0.051	0.61
BSD(35)	0.26	0.00	0.052	0.46
BSD(35)	0.26	0.13	0.052	0.52
BSD(35)	0.26	0.26	0.052	0.54
BSD(35)	0.26	0.39	0.052	0.56
BSD(35)	0.26	0.71	0.053	0.57

Table 14: Sensitivity analysis for BSD(40)

Randomization Procedure [RP]	Selection Bias Effect [η]	Linear-Time Trend Bias Effect [θ]	Type I Error Probability [mean]	$P_{RP}(\omega \leq 0.05)$
BSD(40)	0.00	0.00	0.050	0.00
BSD(40)	0.00	0.13	0.050	0.66
BSD(40)	0.00	0.26	0.050	0.67
BSD(40)	0.00	0.39	0.050	0.67
BSD(40)	0.00	0.71	0.050	0.69
BSD(40)	0.04	0.00	0.050	0.42
BSD(40)	0.04	0.13	0.050	0.52
BSD(40)	0.04	0.26	0.050	0.58
BSD(40)	0.04	0.39	0.050	0.64
BSD(40)	0.04	0.71	0.050	0.67
BSD(40)	0.09	0.00	0.050	0.47
BSD(40)	0.09	0.13	0.050	0.51
BSD(40)	0.09	0.26	0.050	0.52
BSD(40)	0.09	0.39	0.050	0.59
BSD(40)	0.09	0.71	0.050	0.64
BSD(40)	0.14	0.00	0.051	0.51
BSD(40)	0.14	0.13	0.051	0.52
BSD(40)	0.14	0.26	0.051	0.52
BSD(40)	0.14	0.39	0.051	0.56
BSD(40)	0.14	0.71	0.051	0.61
BSD(40)	0.26	0.00	0.052	0.46
BSD(40)	0.26	0.13	0.052	0.52
BSD(40)	0.26	0.26	0.052	0.54
BSD(40)	0.26	0.39	0.052	0.56
BSD(40)	0.26	0.71	0.052	0.58

Table 15: Sensitivity analysis for MP(3)

Randomization Procedure [RP]	Selection Bias Effect [η]	Linear-Time Trend Bias Effect [θ]	Type I Error Probability [mean]	$P_{RP}(\omega \leq 0.05)$
MP(3)	0.00	0.00	0.050	0.00
MP(3)	0.00	0.13	0.050	1.00
MP(3)	0.00	0.26	0.049	1.00
MP(3)	0.00	0.39	0.047	1.00
MP(3)	0.00	0.71	0.042	1.00
MP(3)	0.04	0.00	0.053	0.00
MP(3)	0.04	0.13	0.053	0.00
MP(3)	0.04	0.26	0.052	0.03
MP(3)	0.04	0.39	0.050	0.47
MP(3)	0.04	0.71	0.044	1.00
MP(3)	0.09	0.00	0.064	0.00
MP(3)	0.09	0.13	0.064	0.00
MP(3)	0.09	0.26	0.062	0.00
MP(3)	0.09	0.39	0.061	0.00
MP(3)	0.09	0.71	0.054	0.18
MP(3)	0.14	0.00	0.083	0.00
MP(3)	0.14	0.13	0.083	0.00
MP(3)	0.14	0.26	0.081	0.00
MP(3)	0.14	0.39	0.079	0.00
MP(3)	0.14	0.71	0.072	0.01
MP(3)	0.26	0.00	0.160	0.00
MP(3)	0.26	0.13	0.160	0.00
MP(3)	0.26	0.26	0.158	0.00
MP(3)	0.26	0.39	0.155	0.00
MP(3)	0.26	0.71	0.143	0.00

Table 16: Sensitivity analysis for MP(4)

Randomization Procedure [RP]	Selection Bias Effect [η]	Linear-Time Trend Bias Effect [θ]	Type I Error Probability [mean]	$P_{RP}(\omega \leq 0.05)$
MP(4)	0.00	0.00	0.050	0.00
MP(4)	0.00	0.13	0.050	1.00
MP(4)	0.00	0.26	0.049	1.00
MP(4)	0.00	0.39	0.047	1.00
MP(4)	0.00	0.71	0.042	1.00
MP(4)	0.04	0.00	0.052	0.00
MP(4)	0.04	0.13	0.052	0.01
MP(4)	0.04	0.26	0.051	0.20
MP(4)	0.04	0.39	0.049	0.77
MP(4)	0.04	0.71	0.044	1.00
MP(4)	0.09	0.00	0.059	0.00
MP(4)	0.09	0.13	0.059	0.00
MP(4)	0.09	0.26	0.058	0.01
MP(4)	0.09	0.39	0.056	0.06
MP(4)	0.09	0.71	0.050	0.52
MP(4)	0.14	0.00	0.071	0.00
MP(4)	0.14	0.13	0.071	0.00
MP(4)	0.14	0.26	0.070	0.00
MP(4)	0.14	0.39	0.068	0.01
MP(4)	0.14	0.71	0.061	0.12
MP(4)	0.26	0.00	0.120	0.00
MP(4)	0.26	0.13	0.119	0.00
MP(4)	0.26	0.26	0.118	0.00
MP(4)	0.26	0.39	0.115	0.00
MP(4)	0.26	0.71	0.106	0.01

Table 17: Sensitivity analysis for MP(5)

Randomization Procedure [RP]	Selection Bias Effect [η]	Linear-Time Trend Bias Effect [θ]	Type I Error Probability [mean]	$P_{RP}(\omega \leq 0.05)$
MP(5)	0.00	0.00	0.050	0.00
MP(5)	0.00	0.13	0.050	1.00
MP(5)	0.00	0.26	0.049	1.00
MP(5)	0.00	0.39	0.048	1.00
MP(5)	0.00	0.71	0.042	1.00
MP(5)	0.04	0.00	0.051	0.00
MP(5)	0.04	0.13	0.051	0.06
MP(5)	0.04	0.26	0.050	0.39
MP(5)	0.04	0.39	0.049	0.84
MP(5)	0.04	0.71	0.043	1.00
MP(5)	0.09	0.00	0.056	0.00
MP(5)	0.09	0.13	0.056	0.01
MP(5)	0.09	0.26	0.055	0.06
MP(5)	0.09	0.39	0.054	0.19
MP(5)	0.09	0.71	0.048	0.68
MP(5)	0.14	0.00	0.065	0.00
MP(5)	0.14	0.13	0.065	0.01
MP(5)	0.14	0.26	0.064	0.03
MP(5)	0.14	0.39	0.062	0.06
MP(5)	0.14	0.71	0.056	0.29
MP(5)	0.26	0.00	0.099	0.01
MP(5)	0.26	0.13	0.098	0.01
MP(5)	0.26	0.26	0.097	0.01
MP(5)	0.26	0.39	0.095	0.02
MP(5)	0.26	0.71	0.087	0.07

Table 18: Sensitivity analysis for EBC(0.67)

Randomization Procedure [RP]	Selection Bias Effect [η]	Linear-Time Trend Bias Effect [θ]	Type I Error Probability [mean]	$P_{RP}(\omega \leq 0.05)$
EBC(0.67)	0.00	0.00	0.050	0.00
EBC(0.67)	0.00	0.13	0.050	0.99
EBC(0.67)	0.00	0.26	0.049	0.99
EBC(0.67)	0.00	0.39	0.048	0.99
EBC(0.67)	0.00	0.71	0.043	0.99
EBC(0.67)	0.04	0.00	0.053	0.00
EBC(0.67)	0.04	0.13	0.052	0.02
EBC(0.67)	0.04	0.26	0.052	0.15
EBC(0.67)	0.04	0.39	0.050	0.46
EBC(0.67)	0.04	0.71	0.045	0.92
EBC(0.67)	0.09	0.00	0.064	0.00
EBC(0.67)	0.09	0.13	0.063	0.00
EBC(0.67)	0.09	0.26	0.062	0.02
EBC(0.67)	0.09	0.39	0.061	0.06
EBC(0.67)	0.09	0.71	0.055	0.31
EBC(0.67)	0.14	0.00	0.082	0.00
EBC(0.67)	0.14	0.13	0.082	0.00
EBC(0.67)	0.14	0.26	0.081	0.01
EBC(0.67)	0.14	0.39	0.079	0.02
EBC(0.67)	0.14	0.71	0.072	0.10
EBC(0.67)	0.26	0.00	0.158	0.00
EBC(0.67)	0.26	0.13	0.157	0.00
EBC(0.67)	0.26	0.26	0.155	0.00
EBC(0.67)	0.26	0.39	0.153	0.01
EBC(0.67)	0.26	0.71	0.142	0.02

Table 19: Sensitivity analysis for CHEN(2, 0.67)

Randomization Procedure [RP]	Selection Bias Effect [η]	Linear-Time Trend Bias Effect [θ]	Type I Error Probability [mean]	$P_{RP}(\omega \leq 0.05)$
CHEN(2, 0.67)	0.00	0.00	0.050	0.00
CHEN(2, 0.67)	0.00	0.13	0.050	1.00
CHEN(2, 0.67)	0.00	0.26	0.049	1.00
CHEN(2, 0.67)	0.00	0.39	0.047	1.00
CHEN(2, 0.67)	0.00	0.71	0.042	1.00
CHEN(2, 0.67)	0.04	0.00	0.055	0.00
CHEN(2, 0.67)	0.04	0.13	0.055	0.00
CHEN(2, 0.67)	0.04	0.26	0.054	0.00
CHEN(2, 0.67)	0.04	0.39	0.052	0.10
CHEN(2, 0.67)	0.04	0.71	0.046	0.92
CHEN(2, 0.67)	0.09	0.00	0.074	0.00
CHEN(2, 0.67)	0.09	0.13	0.074	0.00
CHEN(2, 0.67)	0.09	0.26	0.072	0.00
CHEN(2, 0.67)	0.09	0.39	0.071	0.00
CHEN(2, 0.67)	0.09	0.71	0.063	0.02
CHEN(2, 0.67)	0.14	0.00	0.108	0.00
CHEN(2, 0.67)	0.14	0.13	0.107	0.00
CHEN(2, 0.67)	0.14	0.26	0.106	0.00
CHEN(2, 0.67)	0.14	0.39	0.103	0.00
CHEN(2, 0.67)	0.14	0.71	0.094	0.00
CHEN(2, 0.67)	0.26	0.00	0.246	0.00
CHEN(2, 0.67)	0.26	0.13	0.245	0.00
CHEN(2, 0.67)	0.26	0.26	0.243	0.00
CHEN(2, 0.67)	0.26	0.39	0.239	0.00
CHEN(2, 0.67)	0.26	0.71	0.224	0.00

Table 20: Sensitivity analysis for CHEN(3, 0.67)

Randomization Procedure [RP]	Selection Bias Effect [η]	Linear-Time Trend Bias Effect [θ]	Type I Error Probability [mean]	$P_{RP}(\omega \leq 0.05)$
CHEN(3, 0.67)	0.00	0.00	0.050	0.00
CHEN(3, 0.67)	0.00	0.13	0.050	1.00
CHEN(3, 0.67)	0.00	0.26	0.049	1.00
CHEN(3, 0.67)	0.00	0.39	0.047	1.00
CHEN(3, 0.67)	0.00	0.71	0.042	1.00
CHEN(3, 0.67)	0.04	0.00	0.053	0.00
CHEN(3, 0.67)	0.04	0.13	0.053	0.00
CHEN(3, 0.67)	0.04	0.26	0.052	0.02
CHEN(3, 0.67)	0.04	0.39	0.051	0.31
CHEN(3, 0.67)	0.04	0.71	0.045	0.96
CHEN(3, 0.67)	0.09	0.00	0.068	0.00
CHEN(3, 0.67)	0.09	0.13	0.067	0.00
CHEN(3, 0.67)	0.09	0.26	0.066	0.00
CHEN(3, 0.67)	0.09	0.39	0.064	0.00
CHEN(3, 0.67)	0.09	0.71	0.058	0.14
CHEN(3, 0.67)	0.14	0.00	0.092	0.00
CHEN(3, 0.67)	0.14	0.13	0.091	0.00
CHEN(3, 0.67)	0.14	0.26	0.090	0.00
CHEN(3, 0.67)	0.14	0.39	0.088	0.00
CHEN(3, 0.67)	0.14	0.71	0.080	0.01
CHEN(3, 0.67)	0.26	0.00	0.191	0.00
CHEN(3, 0.67)	0.26	0.13	0.190	0.00
CHEN(3, 0.67)	0.26	0.26	0.188	0.00
CHEN(3, 0.67)	0.26	0.39	0.185	0.00
CHEN(3, 0.67)	0.26	0.71	0.172	0.00

Table 21: Sensitivity analysis for CHEN(4, 0.67)

Randomization Procedure [RP]	Selection Bias Effect [η]	Linear-Time Trend Bias Effect [θ]	Type I Error Probability [mean]	$P_{RP}(\omega \leq 0.05)$
CHEN(4, 0.67)	0.00	0.00	0.050	0.00
CHEN(4, 0.67)	0.00	0.13	0.050	1.00
CHEN(4, 0.67)	0.00	0.26	0.049	1.00
CHEN(4, 0.67)	0.00	0.39	0.048	1.00
CHEN(4, 0.67)	0.00	0.71	0.042	1.00
CHEN(4, 0.67)	0.04	0.00	0.053	0.00
CHEN(4, 0.67)	0.04	0.13	0.053	0.00
CHEN(4, 0.67)	0.04	0.26	0.052	0.08
CHEN(4, 0.67)	0.04	0.39	0.050	0.41
CHEN(4, 0.67)	0.04	0.71	0.045	0.94
CHEN(4, 0.67)	0.09	0.00	0.065	0.00
CHEN(4, 0.67)	0.09	0.13	0.065	0.00
CHEN(4, 0.67)	0.09	0.26	0.064	0.00
CHEN(4, 0.67)	0.09	0.39	0.062	0.02
CHEN(4, 0.67)	0.09	0.71	0.056	0.23
CHEN(4, 0.67)	0.14	0.00	0.086	0.00
CHEN(4, 0.67)	0.14	0.13	0.086	0.00
CHEN(4, 0.67)	0.14	0.26	0.084	0.00
CHEN(4, 0.67)	0.14	0.39	0.082	0.00
CHEN(4, 0.67)	0.14	0.71	0.075	0.04
CHEN(4, 0.67)	0.26	0.00	0.171	0.00
CHEN(4, 0.67)	0.26	0.13	0.170	0.00
CHEN(4, 0.67)	0.26	0.26	0.169	0.00
CHEN(4, 0.67)	0.26	0.39	0.166	0.00
CHEN(4, 0.67)	0.26	0.71	0.154	0.00

Table 22: Sensitivity analysis for CHEN(5, 0.67)

Randomization Procedure [RP]	Selection Bias Effect [η]	Linear-Time Trend Bias Effect [θ]	Type I Error Probability [mean]	$P_{RP}(\omega \leq 0.05)$
CHEN(5, 0.67)	0.00	0.00	0.050	0.00
CHEN(5, 0.67)	0.00	0.13	0.050	1.00
CHEN(5, 0.67)	0.00	0.26	0.049	1.00
CHEN(5, 0.67)	0.00	0.39	0.048	1.00
CHEN(5, 0.67)	0.00	0.71	0.042	1.00
CHEN(5, 0.67)	0.04	0.00	0.053	0.00
CHEN(5, 0.67)	0.04	0.13	0.053	0.01
CHEN(5, 0.67)	0.04	0.26	0.052	0.11
CHEN(5, 0.67)	0.04	0.39	0.050	0.45
CHEN(5, 0.67)	0.04	0.71	0.045	0.93
CHEN(5, 0.67)	0.09	0.00	0.064	0.00
CHEN(5, 0.67)	0.09	0.13	0.064	0.00
CHEN(5, 0.67)	0.09	0.26	0.063	0.01
CHEN(5, 0.67)	0.09	0.39	0.061	0.03
CHEN(5, 0.67)	0.09	0.71	0.055	0.28
CHEN(5, 0.67)	0.14	0.00	0.084	0.00
CHEN(5, 0.67)	0.14	0.13	0.083	0.00
CHEN(5, 0.67)	0.14	0.26	0.082	0.00
CHEN(5, 0.67)	0.14	0.39	0.080	0.01
CHEN(5, 0.67)	0.14	0.71	0.073	0.06
CHEN(5, 0.67)	0.26	0.00	0.164	0.00
CHEN(5, 0.67)	0.26	0.13	0.163	0.00
CHEN(5, 0.67)	0.26	0.26	0.161	0.00
CHEN(5, 0.67)	0.26	0.39	0.159	0.00
CHEN(5, 0.67)	0.26	0.71	0.147	0.01

Table 23: Sensitivity analysis for Wei's urn design UD(0,1)

Randomization Procedure [RP]	Selection Bias Effect [η]	Linear-Time Trend Bias Effect [θ]	Type I Error Probability [mean]	$P_{RP}(\omega \leq 0.05)$
UD(0,1)	0.00	0.00	0.050	0.00
UD(0,1)	0.00	0.13	0.050	0.80
UD(0,1)	0.00	0.26	0.050	0.81
UD(0,1)	0.00	0.39	0.049	0.80
UD(0,1)	0.00	0.71	0.046	0.83
UD(0,1)	0.04	0.00	0.050	0.24
UD(0,1)	0.04	0.13	0.050	0.43
UD(0,1)	0.04	0.26	0.050	0.61
UD(0,1)	0.04	0.39	0.049	0.73
UD(0,1)	0.04	0.71	0.047	0.80
UD(0,1)	0.09	0.00	0.052	0.23
UD(0,1)	0.09	0.13	0.052	0.31
UD(0,1)	0.09	0.26	0.051	0.44
UD(0,1)	0.09	0.39	0.050	0.55
UD(0,1)	0.09	0.71	0.048	0.71
UD(0,1)	0.14	0.00	0.054	0.27
UD(0,1)	0.14	0.13	0.053	0.31
UD(0,1)	0.14	0.26	0.053	0.37
UD(0,1)	0.14	0.39	0.052	0.45
UD(0,1)	0.14	0.71	0.050	0.60
UD(0,1)	0.26	0.00	0.062	0.28
UD(0,1)	0.26	0.13	0.061	0.30
UD(0,1)	0.26	0.26	0.061	0.33
UD(0,1)	0.26	0.39	0.060	0.36
UD(0,1)	0.26	0.71	0.058	0.46

Table 24: Sensitivity analysis for Wei's urn design UD(0,2)

Randomization Procedure [RP]	Selection Bias Effect [η]	Linear-Time Trend Bias Effect [θ]	Type I Error Probability [mean]	$P_{RP}(\omega \leq 0.05)$
UD(0,2)	0.00	0.00	0.050	0.00
UD(0,2)	0.00	0.13	0.050	0.80
UD(0,2)	0.00	0.26	0.050	0.81
UD(0,2)	0.00	0.39	0.049	0.81
UD(0,2)	0.00	0.71	0.046	0.83
UD(0,2)	0.04	0.00	0.050	0.24
UD(0,2)	0.04	0.13	0.050	0.43
UD(0,2)	0.04	0.26	0.050	0.61
UD(0,2)	0.04	0.39	0.049	0.73
UD(0,2)	0.04	0.71	0.047	0.80
UD(0,2)	0.09	0.00	0.052	0.24
UD(0,2)	0.09	0.13	0.052	0.31
UD(0,2)	0.09	0.26	0.051	0.44
UD(0,2)	0.09	0.39	0.050	0.55
UD(0,2)	0.09	0.71	0.048	0.70
UD(0,2)	0.14	0.00	0.054	0.27
UD(0,2)	0.14	0.13	0.053	0.30
UD(0,2)	0.14	0.26	0.053	0.37
UD(0,2)	0.14	0.39	0.052	0.45
UD(0,2)	0.14	0.71	0.050	0.61
UD(0,2)	0.26	0.00	0.062	0.28
UD(0,2)	0.26	0.13	0.062	0.29
UD(0,2)	0.26	0.26	0.061	0.33
UD(0,2)	0.26	0.39	0.060	0.36
UD(0,2)	0.26	0.71	0.058	0.46

Table 25: Sensitivity analysis for Wei's urn design UD(0,3)

Randomization Procedure [RP]	Selection Bias Effect [η]	Linear-Time Trend Bias Effect [θ]	Type I Error Probability [mean]	$P_{RP}(\omega \leq 0.05)$
UD(0,3)	0.00	0.00	0.050	0.00
UD(0,3)	0.00	0.13	0.050	0.80
UD(0,3)	0.00	0.26	0.050	0.81
UD(0,3)	0.00	0.39	0.049	0.81
UD(0,3)	0.00	0.71	0.046	0.82
UD(0,3)	0.04	0.00	0.050	0.24
UD(0,3)	0.04	0.13	0.050	0.43
UD(0,3)	0.04	0.26	0.050	0.61
UD(0,3)	0.04	0.39	0.049	0.72
UD(0,3)	0.04	0.71	0.047	0.80
UD(0,3)	0.09	0.00	0.052	0.23
UD(0,3)	0.09	0.13	0.052	0.31
UD(0,3)	0.09	0.26	0.051	0.44
UD(0,3)	0.09	0.39	0.050	0.55
UD(0,3)	0.09	0.71	0.048	0.70
UD(0,3)	0.14	0.00	0.054	0.27
UD(0,3)	0.14	0.13	0.054	0.30
UD(0,3)	0.14	0.26	0.053	0.37
UD(0,3)	0.14	0.39	0.052	0.45
UD(0,3)	0.14	0.71	0.050	0.60
UD(0,3)	0.26	0.00	0.062	0.28
UD(0,3)	0.26	0.13	0.061	0.30
UD(0,3)	0.26	0.26	0.061	0.33
UD(0,3)	0.26	0.39	0.060	0.36
UD(0,3)	0.26	0.71	0.058	0.46

Table 26: Sensitivity analysis for Wei's urn design UD(1,1)

Randomization Procedure [RP]	Selection Bias Effect [η]	Linear-Time Trend Bias Effect [θ]	Type I Error Probability [mean]	$P_{RP}(\omega \leq 0.05)$
UD(1,1)	0.00	0.00	0.050	0.00
UD(1,1)	0.00	0.13	0.050	0.79
UD(1,1)	0.00	0.26	0.050	0.80
UD(1,1)	0.00	0.39	0.049	0.80
UD(1,1)	0.00	0.71	0.046	0.82
UD(1,1)	0.04	0.00	0.050	0.29
UD(1,1)	0.04	0.13	0.050	0.45
UD(1,1)	0.04	0.26	0.050	0.62
UD(1,1)	0.04	0.39	0.049	0.73
UD(1,1)	0.04	0.71	0.047	0.79
UD(1,1)	0.09	0.00	0.051	0.28
UD(1,1)	0.09	0.13	0.051	0.36
UD(1,1)	0.09	0.26	0.051	0.47
UD(1,1)	0.09	0.39	0.050	0.57
UD(1,1)	0.09	0.71	0.048	0.71
UD(1,1)	0.14	0.00	0.053	0.32
UD(1,1)	0.14	0.13	0.053	0.35
UD(1,1)	0.14	0.26	0.053	0.41
UD(1,1)	0.14	0.39	0.052	0.48
UD(1,1)	0.14	0.71	0.049	0.62
UD(1,1)	0.26	0.00	0.060	0.33
UD(1,1)	0.26	0.13	0.059	0.34
UD(1,1)	0.26	0.26	0.059	0.37
UD(1,1)	0.26	0.39	0.058	0.40
UD(1,1)	0.26	0.71	0.056	0.49

Table 27: Sensitivity analysis for Wei's urn design UD(1,2)

Randomization Procedure [RP]	Selection Bias Effect [η]	Linear-Time Trend Bias Effect [θ]	Type I Error Probability [mean]	$P_{RP}(\omega \leq 0.05)$
UD(1,2)	0.00	0.00	0.050	0.00
UD(1,2)	0.00	0.13	0.050	0.80
UD(1,2)	0.00	0.26	0.050	0.81
UD(1,2)	0.00	0.39	0.049	0.80
UD(1,2)	0.00	0.71	0.046	0.82
UD(1,2)	0.04	0.00	0.050	0.27
UD(1,2)	0.04	0.13	0.050	0.45
UD(1,2)	0.04	0.26	0.050	0.61
UD(1,2)	0.04	0.39	0.049	0.73
UD(1,2)	0.04	0.71	0.047	0.79
UD(1,2)	0.09	0.00	0.052	0.26
UD(1,2)	0.09	0.13	0.051	0.34
UD(1,2)	0.09	0.26	0.051	0.46
UD(1,2)	0.09	0.39	0.050	0.57
UD(1,2)	0.09	0.71	0.048	0.71
UD(1,2)	0.14	0.00	0.053	0.30
UD(1,2)	0.14	0.13	0.053	0.33
UD(1,2)	0.14	0.26	0.053	0.40
UD(1,2)	0.14	0.39	0.052	0.47
UD(1,2)	0.14	0.71	0.049	0.62
UD(1,2)	0.26	0.00	0.060	0.32
UD(1,2)	0.26	0.13	0.060	0.33
UD(1,2)	0.26	0.26	0.060	0.35
UD(1,2)	0.26	0.39	0.059	0.39
UD(1,2)	0.26	0.71	0.057	0.48

Table 28: Sensitivity analysis for Wei's urn design UD(1,3)

Randomization Procedure [RP]	Selection Bias Effect [η]	Linear-Time Trend Bias Effect [θ]	Type I Error Probability [mean]	$P_{RP}(\omega \leq 0.05)$
UD(1,3)	0.00	0.00	0.050	0.00
UD(1,3)	0.00	0.13	0.050	0.80
UD(1,3)	0.00	0.26	0.050	0.80
UD(1,3)	0.00	0.39	0.049	0.80
UD(1,3)	0.00	0.71	0.046	0.82
UD(1,3)	0.04	0.00	0.050	0.26
UD(1,3)	0.04	0.13	0.050	0.44
UD(1,3)	0.04	0.26	0.050	0.61
UD(1,3)	0.04	0.39	0.049	0.73
UD(1,3)	0.04	0.71	0.047	0.79
UD(1,3)	0.09	0.00	0.052	0.26
UD(1,3)	0.09	0.13	0.051	0.33
UD(1,3)	0.09	0.26	0.051	0.45
UD(1,3)	0.09	0.39	0.050	0.56
UD(1,3)	0.09	0.71	0.048	0.71
UD(1,3)	0.14	0.00	0.053	0.29
UD(1,3)	0.14	0.13	0.053	0.32
UD(1,3)	0.14	0.26	0.053	0.39
UD(1,3)	0.14	0.39	0.052	0.47
UD(1,3)	0.14	0.71	0.050	0.61
UD(1,3)	0.26	0.00	0.061	0.31
UD(1,3)	0.26	0.13	0.061	0.32
UD(1,3)	0.26	0.26	0.060	0.35
UD(1,3)	0.26	0.39	0.060	0.38
UD(1,3)	0.26	0.71	0.057	0.47

Table 29: Sensitivity analysis for Wei's urn design UD(2,1)

Randomization Procedure [RP]	Selection Bias Effect [η]	Linear-Time Trend Bias Effect [θ]	Type I Error Probability [mean]	$P_{RP}(\omega \leq 0.05)$
UD(2,1)	0.00	0.00	0.050	0.00
UD(2,1)	0.00	0.13	0.050	0.79
UD(2,1)	0.00	0.26	0.050	0.79
UD(2,1)	0.00	0.39	0.049	0.79
UD(2,1)	0.00	0.71	0.047	0.81
UD(2,1)	0.04	0.00	0.050	0.30
UD(2,1)	0.04	0.13	0.050	0.46
UD(2,1)	0.04	0.26	0.050	0.62
UD(2,1)	0.04	0.39	0.049	0.73
UD(2,1)	0.04	0.71	0.047	0.79
UD(2,1)	0.09	0.00	0.051	0.30
UD(2,1)	0.09	0.13	0.051	0.37
UD(2,1)	0.09	0.26	0.051	0.48
UD(2,1)	0.09	0.39	0.050	0.58
UD(2,1)	0.09	0.71	0.048	0.71
UD(2,1)	0.14	0.00	0.053	0.34
UD(2,1)	0.14	0.13	0.053	0.37
UD(2,1)	0.14	0.26	0.052	0.43
UD(2,1)	0.14	0.39	0.052	0.49
UD(2,1)	0.14	0.71	0.049	0.63
UD(2,1)	0.26	0.00	0.059	0.35
UD(2,1)	0.26	0.13	0.059	0.36
UD(2,1)	0.26	0.26	0.058	0.39
UD(2,1)	0.26	0.39	0.058	0.42
UD(2,1)	0.26	0.71	0.055	0.50

Table 30: Sensitivity analysis for Wei's urn design UD(2,2)

Randomization Procedure [RP]	Selection Bias Effect [η]	Linear-Time Trend Bias Effect [θ]	Type I Error Probability [mean]	$P_{RP}(\omega \leq 0.05)$
UD(2,2)	0.00	0.00	0.050	0.00
UD(2,2)	0.00	0.13	0.050	0.79
UD(2,2)	0.00	0.26	0.050	0.80
UD(2,2)	0.00	0.39	0.049	0.80
UD(2,2)	0.00	0.71	0.046	0.82
UD(2,2)	0.04	0.00	0.050	0.28
UD(2,2)	0.04	0.13	0.050	0.45
UD(2,2)	0.04	0.26	0.050	0.62
UD(2,2)	0.04	0.39	0.049	0.73
UD(2,2)	0.04	0.71	0.047	0.79
UD(2,2)	0.09	0.00	0.051	0.28
UD(2,2)	0.09	0.13	0.051	0.35
UD(2,2)	0.09	0.26	0.051	0.47
UD(2,2)	0.09	0.39	0.050	0.57
UD(2,2)	0.09	0.71	0.048	0.71
UD(2,2)	0.14	0.00	0.053	0.31
UD(2,2)	0.14	0.13	0.053	0.35
UD(2,2)	0.14	0.26	0.052	0.41
UD(2,2)	0.14	0.39	0.052	0.48
UD(2,2)	0.14	0.71	0.049	0.62
UD(2,2)	0.26	0.00	0.060	0.33
UD(2,2)	0.26	0.13	0.059	0.35
UD(2,2)	0.26	0.26	0.059	0.37
UD(2,2)	0.26	0.39	0.058	0.40
UD(2,2)	0.26	0.71	0.056	0.49

Table 31: Sensitivity analysis for Wei's urn design UD(2,3)

Randomization Procedure [RP]	Selection Bias Effect [η]	Linear-Time Trend Bias Effect [θ]	Type I Error Probability [mean]	$P_{RP}(\omega \leq 0.05)$
UD(2,3)	0.00	0.00	0.050	0.00
UD(2,3)	0.00	0.13	0.050	0.79
UD(2,3)	0.00	0.26	0.050	0.80
UD(2,3)	0.00	0.39	0.049	0.80
UD(2,3)	0.00	0.71	0.046	0.82
UD(2,3)	0.04	0.00	0.050	0.28
UD(2,3)	0.04	0.13	0.050	0.45
UD(2,3)	0.04	0.26	0.050	0.61
UD(2,3)	0.04	0.39	0.049	0.73
UD(2,3)	0.04	0.71	0.047	0.80
UD(2,3)	0.09	0.00	0.051	0.27
UD(2,3)	0.09	0.13	0.051	0.34
UD(2,3)	0.09	0.26	0.051	0.46
UD(2,3)	0.09	0.39	0.050	0.57
UD(2,3)	0.09	0.71	0.048	0.71
UD(2,3)	0.14	0.00	0.053	0.30
UD(2,3)	0.14	0.13	0.053	0.34
UD(2,3)	0.14	0.26	0.053	0.40
UD(2,3)	0.14	0.39	0.052	0.47
UD(2,3)	0.14	0.71	0.049	0.62
UD(2,3)	0.26	0.00	0.060	0.32
UD(2,3)	0.26	0.13	0.060	0.33
UD(2,3)	0.26	0.26	0.059	0.36
UD(2,3)	0.26	0.39	0.059	0.39
UD(2,3)	0.26	0.71	0.056	0.48
